# Infiltration of Proteins in Cholesteric Cellulose Structures

**DOI:** 10.1021/acs.biomac.1c00183

**Published:** 2021-04-26

**Authors:** Livia K. Bast, Konrad W. Klockars, Luiz G. Greca, Orlando J. Rojas, Blaise L. Tardy, Nico Bruns

**Affiliations:** † Adolphe Merkle Institute, University of Fribourg, Chemin des Verdiers 4, 1700 Fribourg, Switzerland; ‡ Department of Pure and Applied Chemistry, University of Strathclyde, Thomas Graham Building, 295 Cathedral Street, Glasgow G1 1XL, United Kingdom; § Department of Bioproducts and Biosystems, School of Chemical Engineering, Aalto University, P.O. Box 16300, 00076 Aalto, Finland; ∥ Departments of Chemical and Biological Engineering, Chemistry, and Wood Science, University of British Columbia, 2360 East Mall, Vancouver, British Columbia V6T 1Z4, Canada

## Abstract

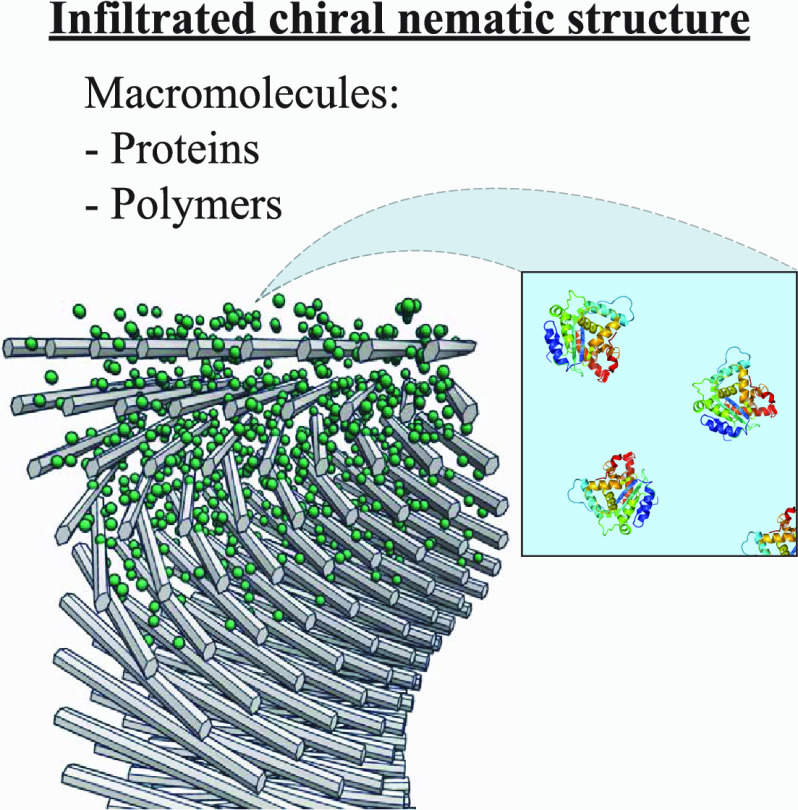

Cellulose nanocrystals (CNCs) can spontaneously self-assemble into chiral nematic (cn) structures, similar to natural cholesteric organizations. The latter display highly dissipative fracture propagation mechanisms given their “brick” (particles) and “mortar” (soft matrix) architecture. Unfortunately, CNCs in liquid media have strong supramolecular interactions with most macromolecules, leading to aggregated suspensions. Herein, we describe a method to prepare nanocomposite materials from chiral nematic CNCs (cn-CNCs) with strongly interacting secondary components. Films of cn-CNCs were infiltrated at various loadings with strongly interacting silk proteins and bovine serum albumin. For comparison and to determine the molecular weight range of macromolecules that can infiltrate cn-CNC films, they were also infiltrated with a range of poly(ethylene glycol) polymers that do not interact strongly with CNCs. The extent and impact of infiltration were evaluated by studying the optical reflection properties of the resulting hybrid materials (UV–vis spectroscopy), while fracture dissipation mechanisms were observed via electron microscopy. We propose that infiltration of cn-CNCs enables the introduction of virtually any secondary phase for nanocomposite formation that is otherwise not possible using simple mixing or other conventional approaches.

## Introduction

**Figure 1:**
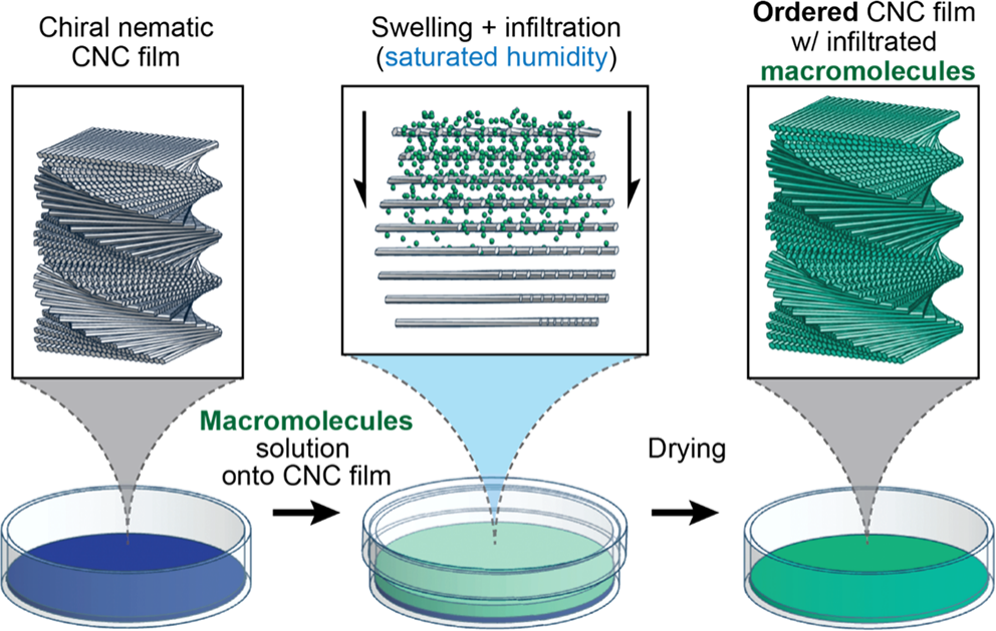
Schematic illustration of the infiltration method used to obtain chiral nematic CNC (cn-CNC) nanocomposites. cn-CNC films (left) were first obtained through evaporation-induced self-assembly (EISA). The films were then infiltrated with strongly interacting or noninteracting macromolecules, leading to simultaneous intercalation of the macromolecules (center) and preservation of the cn structure upon drying (right).

Nature produces hierarchical structures, from the molecular scale to the macroscale. The combination of soft and hard matrices at the nano- and microscales is ubiquitous in natural materials and the evolutionary convergent solution to attain strong and tough materials. Typical examples are bones,^1,2^ seashells, and crustaceans.^3^ The latter consist of fibrils arranged in layers stacked in a helix. Such chiral nematic (cn) structures (Bouligand, plywood, helicoidal, or cholesteric structures) are observed in many organisms.^3−6^ Within each layer, the fibrils are ordered in parallel, but their direction rotates by a fixed angle between the layers (Figure 1).^5,7^ Several synthetic materials have been made using nanocompositing routes to create cn composites with higher strength and toughness than the individual parts they form.^5,8,9^ In the past decade, cellulose nanomaterials, extracted from the cell walls of plant fibers, have been investigated in such contexts.^10^ This is because such fibers are inherently stiff and strong. As such, nanocomposite filaments from cellulose formed with proteins display a tensile strength and Young’s modulus of up to 1 and 55 GPa, respectively.^11^ If cellulose nanofibers are subjected to acid hydrolysis, to remove the disordered cellulosic domains, cellulose nanocrystals (CNCs) are obtained. CNCs possess significantly higher crystallinity than cellulose nanofibers and are therefore inherently stiff (*E*_A_ > 140 GPa) and strong (tensile strength up to 7 GPa).^10,12,13^ The nanorod aspect ratio, generally above 10, can be tuned depending on their source and the processes used to extract them. Additionally, CNCs are insoluble in most conventional solvents; they have a high thermal decomposition temperature (>200 °C), and their surface chemistry can be easily tailored.^14−17^ Because of their outstanding combination of physical–chemical characteristics, CNCs have been considered as a component of nanocomposites.^18^

In biosynthesized structures, hierarchical architectures are regularly found to increase the toughness.^19,20^ Hierarchically ordered materials further increase toughness by dissipating energy through a highly tortuous fracture path. Cellulose nanocrystals can form cn assemblies, where nanorods are aligned within each pseudo-plane and helicoidally arranged across a given direction normal to these planes. These materials are generally obtained by evaporation-induced self-assembly (EISA).^21−25^ cn-CNC films present helicoidal periodicity, a full rotation described as the pitch, resulting in photonic reflections at or near the visible range. cn-CNC structures have been studied to determine the effect of external stimuli on their reflection properties^26,27^ and the effect of the surface on which they are assembled.^28^ The potential to improve toughness through these architectures has been recently highlighted in porous chiral CNC nematic assemblies^29^ or in mixtures of nanoparticles of different sizes.^30^ The reflection property of cn-CNC films is correlated with the nanostructures they form as well as their homogeneity.^31,32^ The reflected colors can be substantially altered and red-shifted using polymers to “stretch” the helices into larger structures^32−35^ or nanoparticles interacting with external fields.^36^ The theory formulated by De Vries^37^ allows for associating the optical rotation with the pitch of the CNC helix (eq 1)
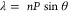
1where λ is the reflected light wavelength normal to the CNC film plane, *n* is the average refractive index of the film, *P* is the pitch size, and *θ* is the angle of incidence to the plane.

Thus far, the formation of cn-CNC nanocomposite films has been achieved by addition of macromolecular compounds or nanoparticles to aqueous CNC suspensions, prior to EISA. Species considered so far have included poly(ethylene glycol) (PEG), poly(vinyl alcohol) (PVA), glucose, and nanoparticles such as gold nanorods.^32−34,38−42^ The list is currently restricted to macromolecules that do not induce a significant aggregation to CNCs, thus maintaining their ability to form cn structures by self-assembly. Importantly, if a macromolecule displays strong interactions with CNCs in a cn assembly, energy dissipation during fracture propagation is expected to substantially increase. Unfortunately, the presence of macromolecules with a high affinity to CNCs leads to aggregation in suspension; hence, no long-range order is formed through EISA. To address this issue, we evaluated a method to infiltrate cn-CNC films with macromolecules, which would otherwise promote a rapid and strong CNC aggregation in suspension (Figure 1). We note that swelling and infiltration of cn-CNC films have been previously attempted using small molecules (ammonium hydroxide,^43^ 2,2-azobis(2-methylpropionitrile),^44,45^ monomer solution cured to poly(dodecanediol-*co*-citrate) post infiltration^46^). However, infiltration of swollen chiral nematic networks followed by dewatering (i.e., drying) is shown here to yield cn-CNC nanocomposite films with up to 50 wt % of an additive. This enables the chiral nematic order to be conserved, even if the latter molecules would otherwise lead to rapid, nonordered aggregation from fully dispersed CNCs. This specially applies to proteins,^11^ polymers,^47,48^ and salts (CaCl_2_, etc.).^49−51^

To demonstrate our running hypothesis, we chose two sets of macromolecules with distinct properties: (1) PEGs that do not interact strongly with CNCs or lead to aggregation in aqueous suspension,^32,33^ and (2) three well-defined structural proteins, all of which are known to cause aggregation when mixed with CNCs but have a high potential to increase the strength and stiffness of composites. (1) PEG was chosen to evaluate the molecular weight (MW) dependency on successful infiltration, and (2) the proteins were chosen to illustrate the benefits over conventional mixing. To this end, we used PEG molecules with MWs ranging from 10 to 1000 kDa, wherein the optomechanical properties of cn-CNC–PEG composites are well reported^32,33,52^ as a function of the fraction of PEG, and bovine serum albumin (BSA, MW = 66.5 kDa,^53^), silk sericin (SS, MW ∼ 15–75 kDa^54^), and silk fibroin (SF, MW ∼ 350 kDa^55^). While BSA is a globular protein of interest due to its wide availability, SS and SF are structural proteins associated with the high strength and toughness of silk.^56^ The latter is a fibrillar protein with a particularly high affinity for cellulose nanofibers.^11^ Finally, we evaluated the tensile strength of PEG- and protein-infiltrated films to extract strength, toughness, and viscoelastic response under tension. The data obtained was critically analyzed to gain insights into the extent of infiltration and its effects on the hierarchical structures. We show that a range of structures can be produced, containing both a chiral nematic order and, potentially, a secondary phase with macromolecules in the network upon infiltration. Beyond the methodology proposed herein, we expect that our results will pave the way toward a better understanding of the interaction of cellulosic nanomaterials with strongly interacting compounds.

## Experimental Section

### Materials

Concentrated slurries of cellulose nanocrystals (CNCs, CAS no. 7789-20-0) were obtained from the University of Maine, through the Process Development Center. They were produced by the USDA’s Forest Product Laboratory in Madison, WI, using sulfuric acid hydrolysis of wood pulp. The stock CNC suspension (10.4 wt %) was diluted with Milli-Q water (Millipore, Synergy UV).

Poly(ethylene glycols) (PEGs) of four different molecular masses were used. The sample of 10 kDa was purchased from Fluka, and those of 100, 400, and 1000 kDa were from Sigma-Aldrich. Solid 0.35 mm thick polyamide (nylon 6) sheets were purchased from Merck. Solvents (methanol (MeOH), absolute ethanol (EtOH), glacial acetic acid, and formic acid (FA)) were purchased from Sigma-Aldrich and used without further purification. Ultrapurified water (Milli-Q water, Merck KGaA, Darmstadt, Germany) was used to prepare infiltration solutions of protein and to degum silk cocoons. Lyophilized powders of bovine serum albumin (BSA), Coomassie Brilliant Blue G-250, sodium carbonate (Na_2_CO_3_), calcium chloride (CaCl_2_), poly(ethylene glycol) (PEG, MW 35 kDa), and ethylene glycol were also purchased from Sigma-Aldrich (Buchs, Switzerland) and used without further purification. Dried silkworm cocoons (species *Bombyx mori*) were purchased from Wollspinnerei Vetsch (Pragg-Jenaz, Switzerland).

### Methods

#### Extraction of Silk Fibroin and Silk Sericin

Based on the standard protocol of Rockwood et al.,^57^ silk fibroin was extracted from dried silk cocoons. The extraction method of silk fibroin and silk sericin is thoroughly described in the Supporting Information ("Extraction of Silk Fibroin and Silk Sericin").

#### Preparation of CNC and Protein Films

Free-standing CNC films were prepared from a 5.5 wt % CNC suspension. The suspension was poured onto a flat polyamide surface that was previously modified by adhering a paraffin (Parafilm M) frame that set physical boundaries for the cast suspension, such that the three-phase contact line was pinned along the frame edges, for an effective areal density of 0.33 mL cm^–2^. CNC films were prepared through EISA in controlled ambient conditions (50% relative humidity (RH) and 23 °C).

Alternative CNC films were cast for Figure S2, and the corresponding experimental work can be found in the Supporting Information (“Alternative CNC Films”).

Protein films, used as reference, were solvent-cast from a 15 wt % protein solution onto the lid of a well-plate (TPP Techno Plastic Products AG, Trasadingen, Switzerland), dried in a fume hood overnight, and carefully removed with a spatula. One of the dried SF films was placed in MeOH for 20 min to induce β-sheet formation.

#### Infiltration of CNC Films with Poly(ethylene glycol) and Proteins

Dried CNC films were infiltrated with aqueous solutions of a given concentration of PEG, BSA, SF, or SS (Table 1). Denatured SF infiltration solutions were prepared by dissolving silk fibroin fibers (SFFs) in formic acid at the desired concentration and stirred for 1 h. A reference infiltration with water (Milli-Q) was also performed. An infiltration ratio of (dry CNC) film mass-to-volume (infiltration solution) equivalent to 1:10 was used. First, a flat and rigid polyamide surface was placed in a plastic container (diameter: 7–8.5 cm, height 5.8 cm, 230 mL, purchased from Huhtamäki) with wet tissue paper, and a dried cn-CNC film was placed lying flat on the polyamide surface. Then, the infiltration solution was carefully added onto the top side of the film, avoiding spilling over the edges onto the polyamide surface. The solution completely covered the surface of the sample. The lid to the container was then sealed tight using a mechanically locked lid, creating an atmosphere of 100% relative humidity and enabling solute infiltration into the CNC film for at least 24 h. Afterward, the respective swollen CNC film (together with the polyamide surface) was carefully removed from the container and allowed to dry overnight in a fume hood. A higher solution concentration led to a higher solute-to-CNC ratio in the dried, postinfiltration films. To elucidate the level of isolation of the 100% relative humidity atmosphere, the lid of one container was resealed after removing the film. When the lid was subsequently opened after one month, the paper towel was still saturated with water.

**Table 1:** CNC Films Infiltrated with Macromolecular Additives (PEG and Protein Solutions) at Given Infiltration Concentrations

sample code	additive	additive solvent^a^	*c* (additive), wt %	CNC/additive weight ratio in dry film
I-REF-CNC	none	water		100:0
I-PEG_10_-40	PEG, 10 kDa	water	4	100:40
I-PEG_100_-40	PEG, 100 kDa	water	4	100:40
I-PEG_400_-20	PEG, 400 kDa	water	2	100:20
I-PEG_400_-40	PEG, 400 kDa	water	4	100:40
I-PEG_1000_-20	PEG, 1000 kDa	water	2	100:40
I-PEG_1000_-40	PEG, 1000 kDa	water	4	100:40
I-BSA-10	BSA	water	1	100:10
I-BSA-25	BSA	water	2.5	100:25
I-BSA-50	BSA	water	5	100:50
I-BSA-100	BSA	water	10	100:100
I-SF-25	SF	water	2.5	100:25
I-SF-40	SF	water	4	100:40
I-SF-25-denat	SF	formic acid^b^	2.5	100:25
I-SF-10-β	SF	post treatment with MeOH for 20 min and 24 h	1	100:10
I-SF-25-β	SF		2.5	100:25
I-SS-10	SS	water	1	100:10
I-SS-40	SS	water	4	100:40

a Water was used as a solvent to prepare a solution of additives, unless otherwise mentioned.

b Formic acid was used as a chaotropic agent to dissolve silk fibroin.

Conformational changes (β-sheet formation) of SF infiltrated in the CNC films were induced by treatment with methanol (20 min to 24 h). A reference CNC film was treated the same way, without observing any differences upon naked-eye inspection.

#### Preparation of CNC Films with PEG or Proteins by Mixing

As a reference, composite CNC films were prepared with additive solutions premixed with the CNC suspension, prior to EISA. The CNC suspension was mixed with the solution (Table 2) and Milli-Q water (Millipore, Synergy UV) to obtain the given CNC/additive ratio. Films were then cast according to the previous procedure, with the exception that the areal density was fixed at 0.22 mL cm^–2^ for samples M-PEG_10_-40, M-PEG_100_-40, M-PEG_400_-20, and M-PEG_1000_-20.

**Table 2:** CNC Films Prepared by Mixing the CNC Suspension with PEG or Protein Solution

sample code	additive	CNC/additive
REF-CNC		100:0
M-PEG_10_-40	PEG, 10 kDa	100:40
M-PEG_100_-40	PEG, 100 kDa	100:40
M-PEG_400_-20	PEG, 400 kDa	100:20
M-PEG_1000_-20	PEG, 1000 kDa	100:20
M-BSA-25	BSA	100:25
M-SS-25	SS	100:25

#### UV–Vis Spectroscopy

All cast CNC films were analyzed with a UV–vis–near-infrared (NIR) spectrophotometer (Agilent Cary 5000) operated in the transmission mode. The films adhered to the Petri dishes (only shown in Figure S2) were measured thrice at different positions within the central blue region of the film. The free-standing films were measured at four positions per film, except for the REF-SF, M-PEG_10_-40, M-PEG_100_-40, M-PEG_400_-20, and M-PEG_1000_-20 samples, which were measured at a single position. All films were measured within the central blue region of the film. Due to the change in the lamp in the spectrophotometer, some of the spectra showed a transmission % signal spike from 348 to 350 nm and should be taken as an artifact. This was corrected by adjusting the transmission values at and below 348 nm, using a factor corresponding to the transmission % at 348 nm divided by that at 350 nm.

#### Imaging of CNC Films

Selected CNC films were photographed using a Canon 60D 18.1 megapixel camera (6000 × 4000 resolution). The films were photographed using the setup described in a previous work.^58^ The brightness and contrast of the images were adjusted using Adobe Photoshop software. In addition, low-magnification (8×) and high-resolution (6000 × 4000) microscope images were acquired for some films, using an Olympus SZX10 optical microscope used in the reflection mode.

#### Controlled Fracturing and Scanning Electron Microscopy (SEM)

Select dry infiltrated CNC films were fractured according to two different methods. (1) Films were fractured by a tensile zero-span pulling force in an L&W tensile tester operated at 50% humidity (conditioned for 1 day) using a pulling speed of >1 mm s^–1^. (2) Films were bent by hand slowly until completely fractured into two pieces. A 4 nm thick platinum–palladium coating was sputtered onto the fractured films, after which they were imaged in a Sigma Zeiss ULTRA-plus scanning electron microscope. The brightness and contrast of the whole images were adjusted using Adobe Photoshop software. Note that due to the low pitch in the cn-CNC films, some burning artifacts may have occurred, such as minor collapsing of protruding CNCs (aligned perpendicular to the cross-section plane).

#### Coomassie Staining of Protein-Infiltrated CNC Films

A staining and destaining solution prepared following Lämmli^59^ was used to treat the protein-infiltrated cn-CNC films. Noninfiltrated CNC films were stained and used as reference. A detailed description of the above procedure is included in the Supporting Information ("Coomassie Staining of Protein-Infiltrated CNC Films"). Coomassie-stained films were cut with a razor blade to expose cross sections perpendicular to the film plane. The films were imaged in an Olympus BX53M optical microscope equipped with an Olympus DP74 camera, in both reflection and transmission modes. Under the illumination conditions used for the transmission mode imaging, the photodetectors were fully saturated when imaging the unstained references of CNC films I-REF-CNC and I-SF-25, which indicate their high transversal transmission prior to staining.

#### Mechanical Properties

Tensile tests were performed using an Allround Line static material testing machine (Z010, ZwickRoell GmbH & Co. KG, Ulm, Germany) equipped with a 200 N Xforce HP load cell. In accordance with ASTM D 1708-18, at least five specimens of each composition (unless otherwise mentioned) were tested at 25 °C. The test specimens were cut from PEG or protein-infiltrated CNC films into rectangular shapes using a surgical scalpel with a fresh and clean blade (4 GS/S, blade no. 20, Swann-Morton, Sheffield, U.K.). The thicknesses of the test specimens were determined as an average of three random locations using an absolute digimatic 2 μm. The widths of the test specimen (3–4 mm) were determined using a digital ABS AOS caliper (Mitutoyo, Kawasaki, Japan). A traverse speed of 0.5 mm min^–1^ was chosen and a maximum force of 8 kN. Test specimens were equilibrated in a desiccator at 23 ± 2 °C and 55 ± 3% RH for 5 days. The data was corrected for biases as induced by film bending in the very low elongation regime (< 0.5%). The Young’s modulus was calculated from the initial linear region of the stress–strain curve (*R*^2^ ≥ 0.99). The area under the curve was calculated as toughness, and the ultimate tensile strength was defined as the maximum stress at break. It is important to note that all samples were fragile and brittle. Also, due to the limited film material, it was not possible to test all five specimens of the protein-infiltrated composites. The standard deviation was calculated from five specimens, except for I-PEG_10_-40 and I-BSA-50 (*n* = 1), I-BSA-25 and I-SS-25 (*n* = 2), and I-SF-25 (*n* = 3).

#### Fourier Transformation Infrared (FT-IR) Spectroscopy

FT-IR spectra were recorded using a Spectrometer 65 (PerkinElmer, Waltham). Samples were measured in the transmission mode, while a background spectrum was collected from air. For each measurement, 50 scans with a resolution of 4 cm^–1^ were averaged, and spectra were collected from both sides (upper and bottom sides) of the infiltrated and reference films. Data analysis was performed using the software Spectrum 6.

## Results and Discussion

### Infiltration

The given macromolecules (PEGs and proteins) were infiltrated into cn-CNC films prepared through evaporation-induced self-assembly (EISA). First, the cn-CNC films were swollen with the infiltrating aqueous solutions (Figure 1) and kept in a 100% humidity environment for 24 h. Then, upon drying, it was assumed that the given additive was fully contained in the cn-CNC films, which allowed the selection of the CNC-to-additive mass ratio in the final film, depending on the additive concentration in the infiltrating solution. No washing steps were performed post infiltration. To avoid redispersion of the CNC film and to keep it above the gelling point, the concentration of CNCs was kept above 10 wt % by limiting the infiltration solution addition to 10-fold mass with respect to the original weight of the CNC film. Note that higher additive concentration in the infiltration solution led to lower flowability, which affected infiltration, as described in subsequent sections.

The reflection color of the films (UV–vis spectroscopy) allows examination of the effect of the infiltrating additive in increasing the pitch of the cn structure. The CNCs used here formed cn-CNC films with an average pitch of ca. 156 nm (Figure S1), leading to photonic reflections in the UV range according to eq 1. This is a smaller pitch to what is generally reported. Such films were used for all infiltrations to better emphasize the lower-molecular-weight limits of the macromolecular additives used. The absorption bands corresponding to specific reflections resulting from chiral nematic structures occurred at <400 nm in our CNC films, while above this absorption band, non-Bragg reflection (herein reported at 800 nm) corresponded to nonspecific reflections.

**Figure 2:**
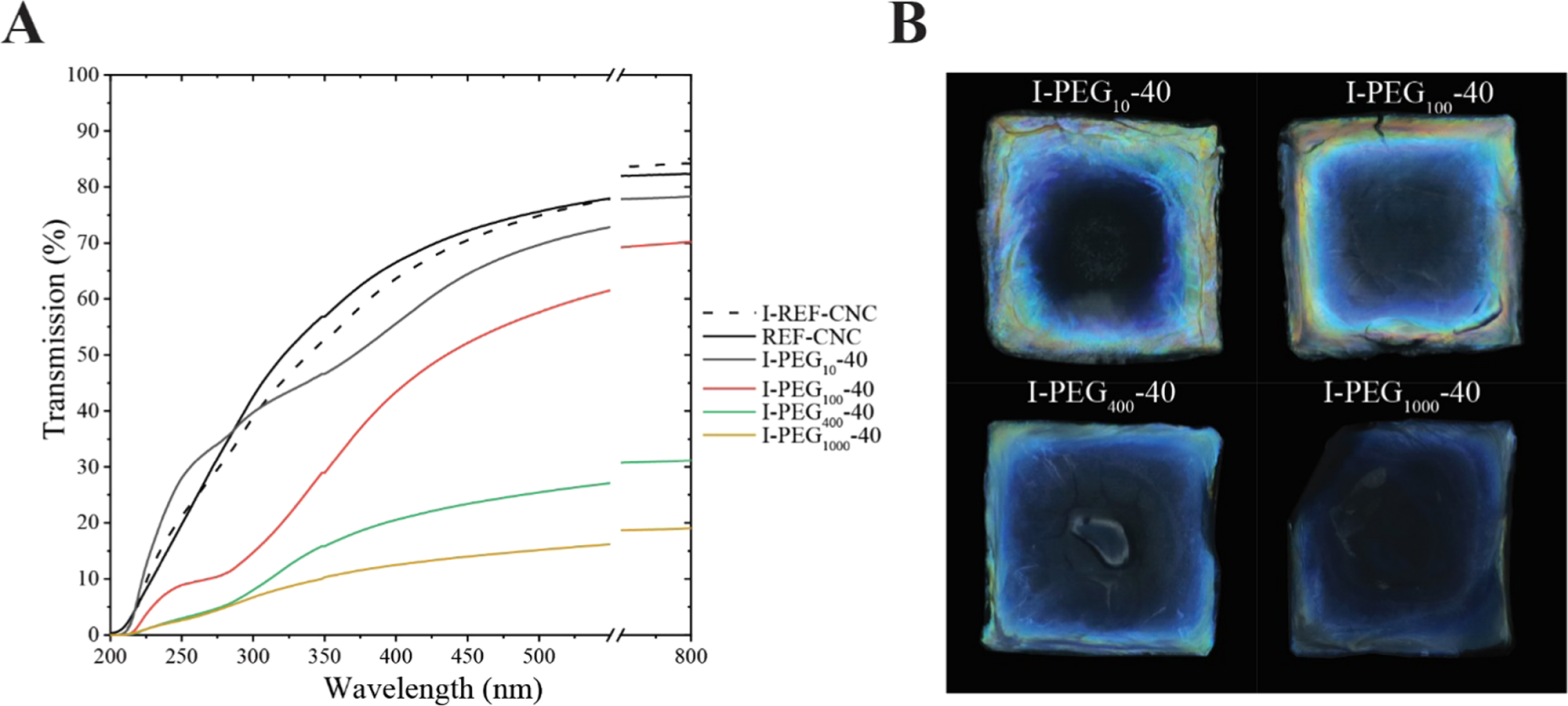
(A) UV–vis transmission spectra of a cn-CNC film (REF-CNC), a cn-CNC film infiltrated with water (I-REF-CNC), and CNC films infiltrated with PEGs of a given molecular mass (kDa indicated in the indices used in the sample’s names). The abscissa (*x*-axis) was broken in the 550–750 nm region to emphasize the red shifts below 550 nm. (B) Photographs of CNC films infiltrated with PEG, taken normal to the film plane and parallel to the light source, and with the infiltrated front facing the viewer.

As a reference, infiltration was performed with an additive-free solution (pure water). This allowed investigating the effect of swelling and redrying on the long-range chiral nematic order of the CNC films. No significant change in the UV–vis spectra was observed (Figure 2A), which suggests that the cn structure in the CNC film was not adversely affected by swelling and redrying. To further show the retention of the cn structure, films were cast through EISA of a different batch of the CNC suspension, generating pitches that reflected light at > 400 nm. Figure S2 shows almost identical UV–vis spectra when comparing a CNC film before and after infiltration (swelling + redrying). Figure S2 also shows optical microscopy images at the same position in the film before and after infiltration, which indicated close similarity. These results indicated that the infiltration procedure caused minimal disruption to the cn structure.

In the existing literature, CNC composites with given macromolecules have been prepared by mixing the components, prior to film casting.^32−34,38,60^ Therefore, for comparative purposes, samples were also prepared by such premixing with a CNC suspension and subsequently cast into films. However, the premixing method limits the formation of the cn order in CNC films during EISA or completely prevents it. This was especially apparent in the present work, when CNCs were mixed with silk fibroin (SF) in suspension (Figure S3), providing a strong justification for the infiltration method introduced in this work.

The nomenclature used to refer to the samples includes reference to the preparation method, either infiltration (I) or premixing (M), the type of macromolecule (PEG, BSA, SS, or SF), and the final concentration of the additive in the dried film relative to the CNC mass, as % dry weight (10, 20, 40, 50, or 100 wt %). Reference samples containing only a single compound are referred to as REF-CNC, REF-BSA, and REF-SF. A REF-CNC film swollen in pure water and then dried again is referred to as I-REF-CNC.

### Infiltration with Poly(ethylene glycol)

**Figure 3:**
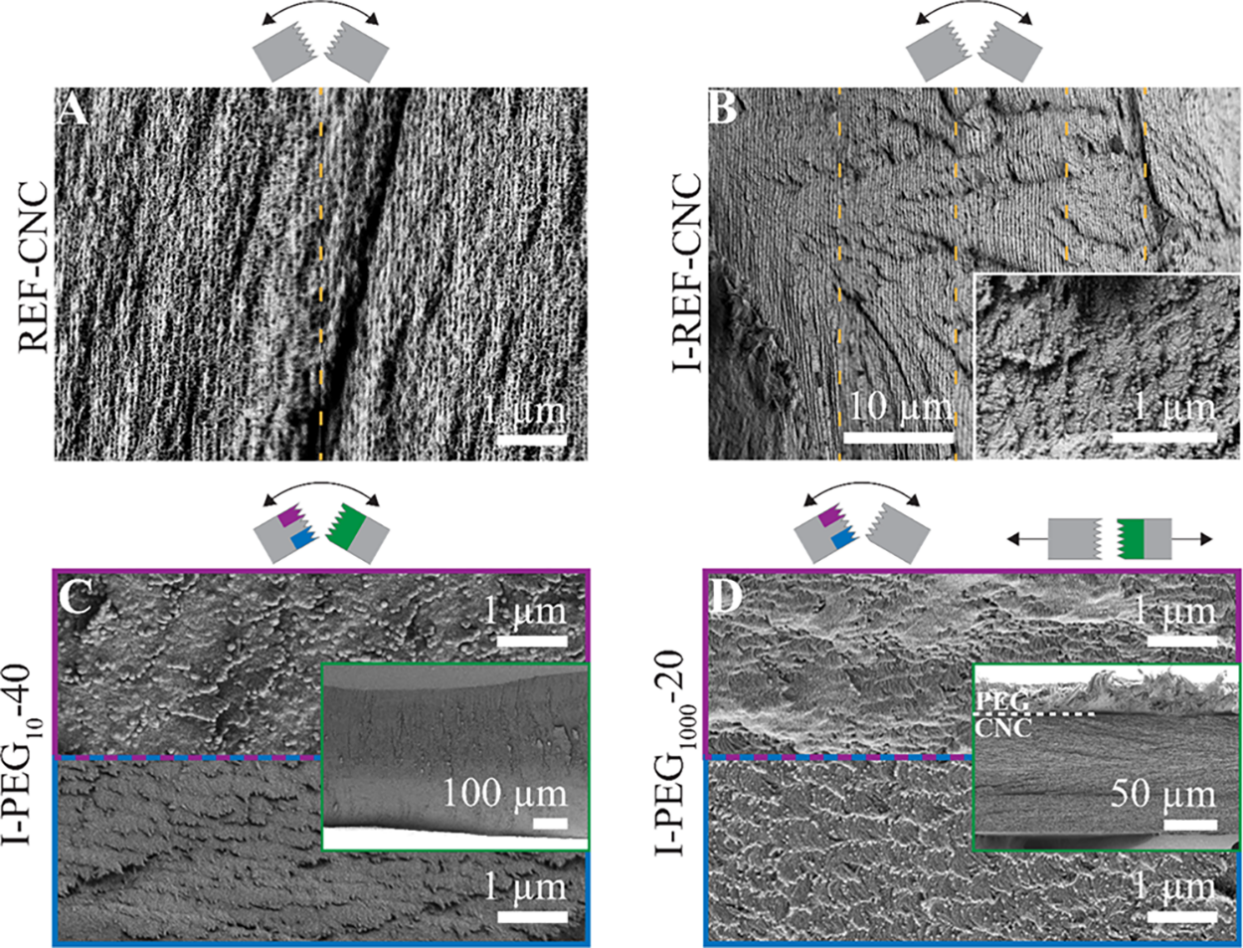
Scanning electron microscopy (SEM) images of CNC films and those infiltrated with PEG of a given molecular mass. The inset of (D) corresponds to the cross section of a film fractured by tensile pulling, where the viewing direction is along the plane. The other images are cross-section images of films fractured by bending, where the viewing direction is oblique to the film plane. (A) Pristine CNC film. (B) CNC film infiltrated with water, i.e., swollen and redried. (C) CNC film infiltrated with 10 kDa PEG. (D) CNC film infiltrated with 1000 kDa PEG. The dashed orange lines correspond to the borderlines of different images, placed together to allow sharp focus despite the tilted surface. Images in purple and blue frames are taken from the topmost and bottom parts of the film, respectively. Green framed insets show the full cross section of the films. These cross sections revealed the presence or absence of macrofractures as well as the specific presence of an upper PEG layer in (D).

**Figure 4:**
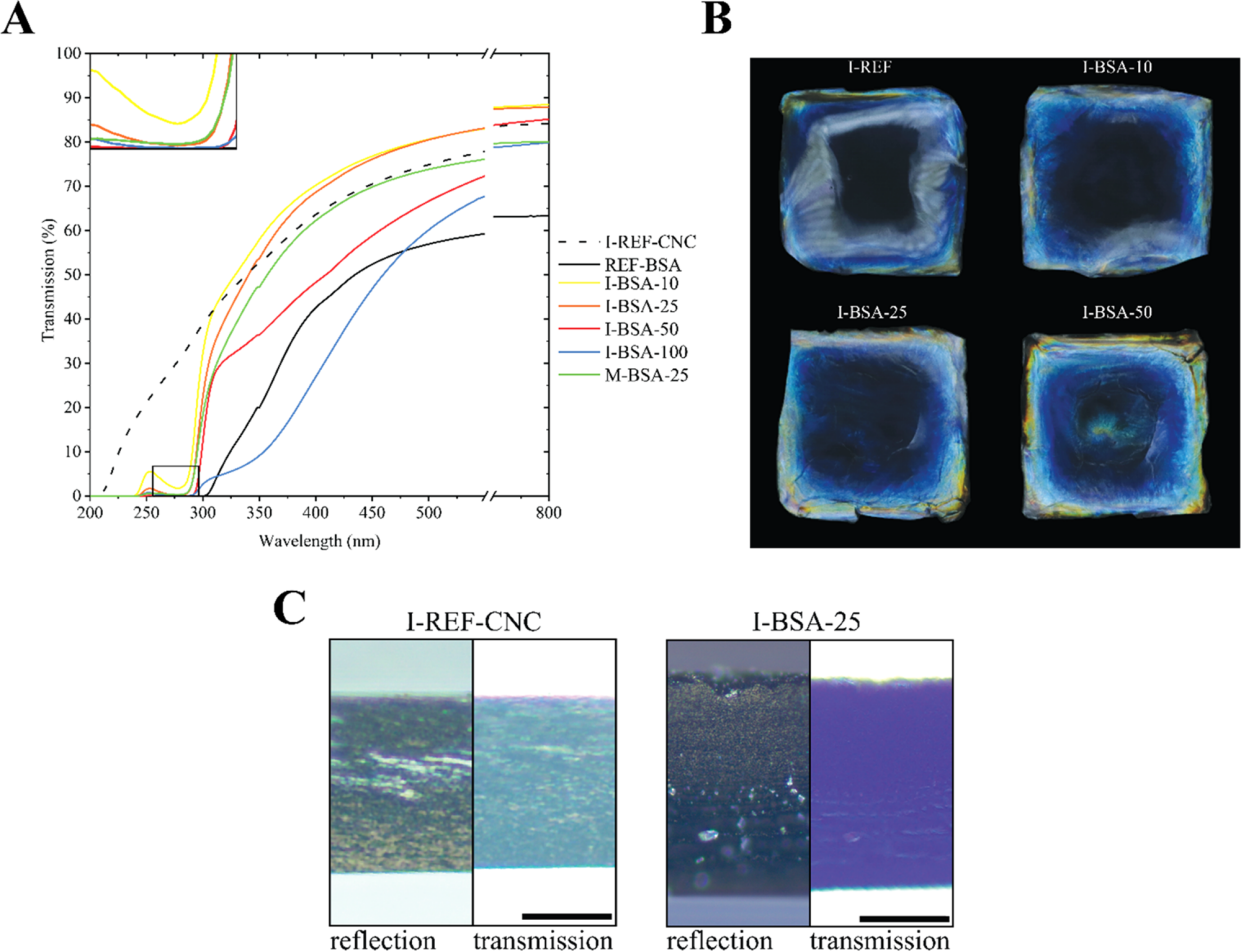
(A) UV–vis transmission spectra of BSA-infiltrated CNC films (I-BSA), and a solvent-cast film of a premixed suspension comprising CNCs and BSA (M-BSA-25). REF-BSA (black line) represents a BSA film without CNCs, and I-REF-CNC (dashed line) represents a cn-CNC film infiltrated with water. The abscissa (*x*-axis) was broken in the 550–750 nm region to emphasize the red shifts below 550 nm. (B) Photos of infiltrated films, taken normal to the film plane and parallel to the light source. The photos were taken with the infiltrated front facing upward. (C) Cross sections of cn-CNC films stained with Coomassie blue, where the left section represents microscopy images taken in the reflection mode and the right section corresponds to microscopy images taken in the transmission mode. Figure S6C shows an unstained version of I-REF-CNC. Please note that the bright areas in the reflection images of all samples are light reflections on the uneven surface of the cross sections. Scale bars correspond to 19.5 μm for the I-REF-CNC film and to 50 μm for the I-BSA-25 film.

We first evaluated the effect of macromolecule molecular mass on diffusion-induced infiltration. Here, poly (ethylene glycol) (PEG) was used as a noninteracting model molecule to estimate the limits of infiltration in terms of molecular weight, so that the information could be translated to the case of the highly interacting macromolecules tested. To this end, four different molecular weights were chosen: PEG_10_, PEG_100_, PEG_400_, and PEG_1000_ (the subscripts refer to the respective molecular mass, kDa). UV–vis spectra show the reflected color of the films as transmission minima (Figure 2A). For the films infiltrated with PEG, a red shift of the reflected color was observed, indicating an increased pitch, given successful infiltration (Figure 2A). This was coupled with a reduction in nonspecific transmittance that was more pronounced with increasing MW. This suggests a reduced homogeneity in samples infiltrated with PEG of larger molecular weights. Photos of the films (Figure 2B) show that the films infiltrated with PEG_10_ and PEG_100_ were slightly red-shifted compared to the control sample in Figure 4B. Meanwhile, a more limited effect was observed for PEG_400_ and PEG_1000_. Accordingly, I-PEG_400_-40 and I-PEG_1000_-40 (Figure 2A) display a sharp decrease in nonspecific transmittance, leading to a widening of the reflection band, indicating the reduced red shift that was also observed in the photographs (Figure 2B). Figure S4A shows photographs of a CNC film infiltrated with 2% PEG instead of 4%, I-PEG_1000_-20, imaged both from the top and the bottom side. The bottom side was more reflective, suggesting a limited additive diffusion or infiltration, where a PEG-only layer might form on top of the cn-CNC film, since reflections going through an opaque PEG layer would be dimmer. Details related to these observations are covered in the discussion related to Figure 3D.

The UV–vis spectra of films prepared by premixing instead of infiltration had a high nonspecific transmittance, even for samples infiltrated with PEG_1000_ (Figure S4B). Furthermore, a narrower reflection bandwidth was observed compared to the infiltrated systems, indicating an increased homogeneity in the dispersion of PEG in the films. Premixing larger fractions of PEG, 40 instead of 20, resulted in red-shifted films, according to a previous work.^32^

For the CNCs used herein, a 10-fold out-of-plane swelling upon infiltration of the cn-CNC system was estimated.^61,62^ Based on the reduced nonspecific transmittance and the widening of the reflection band, a threshold between 400 and 1000 kDa was identified for a successful infiltration. Note that due to the highly hygroscopic nature of PEG, some of the red shift observed may be assigned to water sorption with PEG, which swelled the film and expanded the pitch distance. However, in the context of this work and since exact quantification of the red shift was not needed, decoupling of the two effects (shift caused by PEG infiltration and the water-associated swelling) was not attempted. Moreover, we note that any layer of noninfiltrated, hygroscopic macromolecules adsorbed on the surface of the CNC film does not lead to the increase of the cn pitch. Finally, flowability played an important role; for instance, PEG_1000_ did not infiltrate efficiently, even at a dilute concentration (2 wt %), given that a sol–gel transition into a highly viscous gel occurs for PEG 1000 kDa even at such low concentrations. Overall, the results suggest that infiltration did occur for all PEGs except for PEG_1000_ and only partial infiltration took place for PEGs of MW > 100 kDa.

Infiltrated cn-CNC films were fractured and analyzed by scanning electron microscopy (SEM). Sample films were bent until a fracture or a tear developed, and the obtained cross sections were imaged at oblique angles to the film plane. Figure 3A shows the presence of cn ordering in a pristine CNC film, while Figure 3B shows a similar order in a CNC film swollen with water and subsequently dried. The inset (Figure 3B) shows a magnified image of the cn structure. Figure S5A,B further shows examples of the pristine and swollen-dried CNC films. The SEM images indicate that the cn order is preserved through the infiltration method, as was shown before by microscopy and UV–vis spectroscopy (Figures S2 and 2A). The inset in Figure 3C reveals a smooth fracture of a film infiltrated with PEG 10 kDa, with no visible macroscopic PEG domains. The image in the blue frame (Figure 3C) shows conserved cn domains, with stretched Bouligand structures along the elongated fractures.

A second fracturing method was used for the PEG-infiltrated films. The span between clamps in a zero-span tensile device was 600 μm, and a rapid tension-induced fracture was obtained. This second fracture type highlights the viscoelastic response of the composite matrix, while the primary tear/bend fracture facilitates visualization of the homogeneity of the long-range order and of the infiltration in the film. In contrast to PEG_10_, films infiltrated with PEG_1000_ showed two distinct domains (separated by a dashed line in the image) in the tensile-pulled sample, corresponding to CNC and PEG, indicating no significant infiltration (Figure 3D, inset). Bouligand structures were maintained, and there was no clear indication of infiltration in the lower part of the film infiltrated with PEG_1000_ (Figure 3, bottom). However, there was partial infiltration at the very top of the interface between the additive and cn-CNC film (Figure 3, top), as indicated by the blurred contour of the stretched Bouligand structures. The images in Figure 3D highlight the difference in qualitative reflectivity observed between the top and bottom sides of the photo of PEG_1000_ in Figure S4A. Tensile pulling of CNC films infiltrated with PEG_10_ and PEG_100_ resulted in alignment of the nanocrystals along the strain direction (Figure S5C_2_,D) as was previously shown for PVA–CNC nanocomposites.^33^

### Protein Infiltration

So far, we have shown that full infiltration with PEG occurs at up to MW of 100 kDa. PEG 1000 kDa corresponded to the upper limit for partial infiltration. Herein, we discuss the infiltration of proteins with molecular weights < 400 kDa using the same method as for the PEG infiltration (Figure 1). Bovine serum albumin (BSA), which is a globular protein, and two structural proteins, silk sericin (SS) and silk fibroin (SF), were chosen to investigate their impact on the structure, optical, and mechanical properties of the cn-CNC films. Particularly, SF protein is well-known for its ability to form a high-strength proteinaceous material and has a favorable affinity to nanocellulose.^11,63,64^

**Figure 5:**
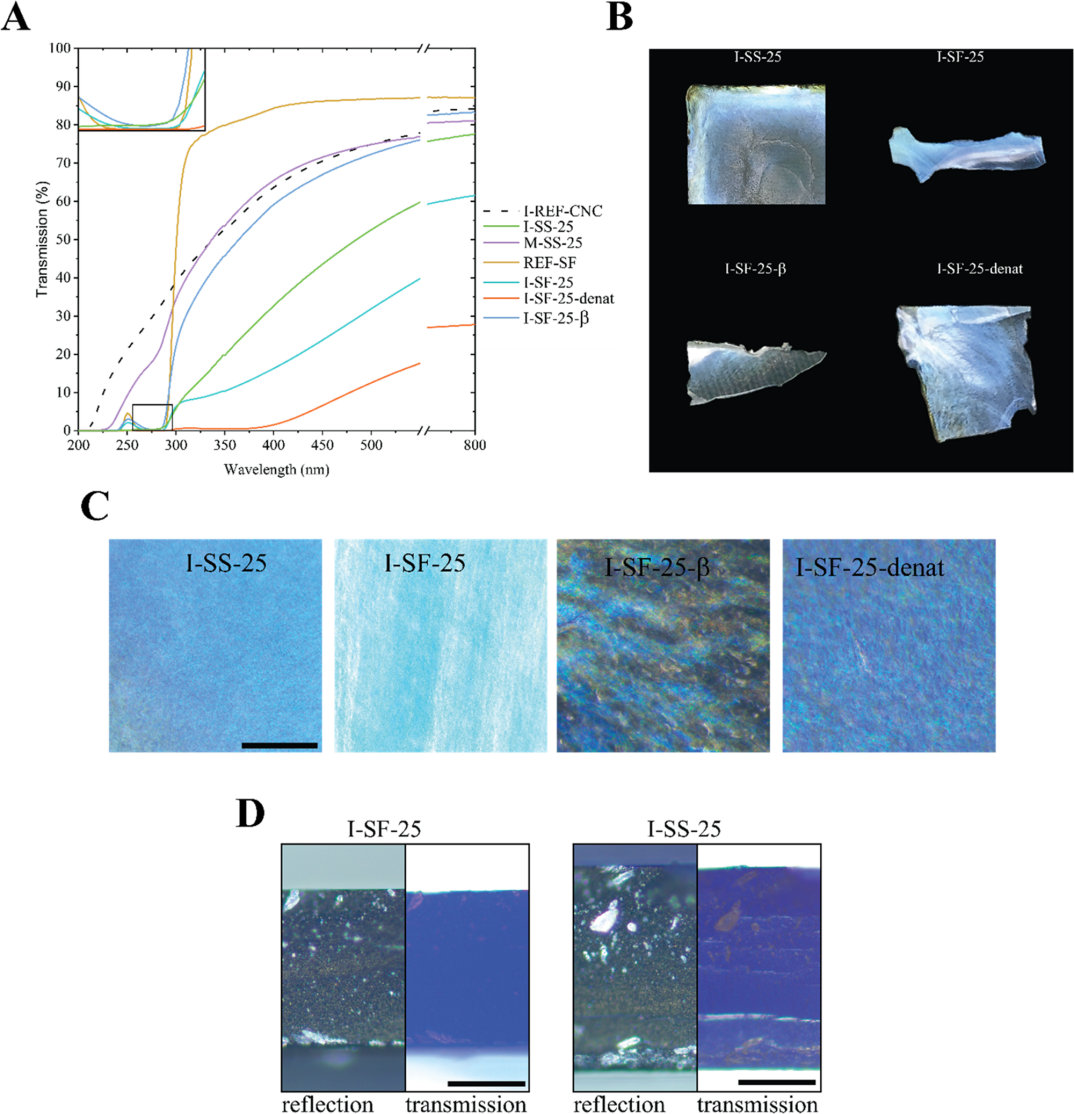
(A) UV–vis transmission spectra of cn-CNC films infiltrated with silk sericin and silk fibroin (I-SS-25 and I-SF-25) and a solvent-cast film of a premixed CNC suspension containing SS (M-SS). I-REF-CNC (dashed line) represents a cn-CNC film infiltrated with water. Prior to infiltration, SF was denatured in formic acid (I-SF-25-denat). Post-treatment of SF-infiltrated CNC films with methanol induced β-sheet formation of the protein (I-SF-25-β). A variation of concentrations of SS and SF is presented in Figure S6. The abscissa (*x*-axis) was broken in the 550–750 nm region to emphasize the red shifts below 550 nm. (B) Photographs of CNC films infiltrated with SS and SF, taken normal to the film plane and parallel to the light source. Photos were taken from fragments from tensile stress measurements. (C) Microscopy images of silk-protein-infiltrated CNC films, taken in the reflection mode. The scale bar represents all of the images in (C) and corresponds to 500 μm. (D) Cross sections of infiltrated cn-CNC films stained with Coomassie blue, where the left section corresponds to microscopy images taken in the reflection mode and the right section represents microscopy images taken in the transmission mode. Figure S6C shows an unstained version of I-SF-25. Please note that the bright areas in the reflection images of all samples are light reflections on the uneven surface of the cross sections. Scale bars correspond to 50 μm.

Successful infiltration with protein was expected to produce a red-shifted reflection due to swelling and increased pitch of cn-CNC, as was the case for PEG (Figure 2A). Figure 4A shows that the reflections red-shifted in proportion to the ratio of incorporated BSA. However, the UV adsorption band from proteins (i.e., from their aromatic amino acids) masks this red shift, unless enough protein is infiltrated, as is the case for I-BSA-100 and partially for I-BSA-50. This band is highlighted in the inset of Figure 4A. The same absorption also occurs for SS- and SF-infiltrated films, shown in the inset of Figure 5A. The red shift is more clearly visible when observing photographs of BSA films, where the color slowly red shifts with increasing infiltration concentration (Figure 4B, more pronounced toward the edges of films).

Infiltration with 1–10 wt % (10–100 mg mL^–1^) BSA solution did not lead to a reduction in nonspecific transmittance, which was also the case for CNCs premixed with BSA (Figure 4A). The red shift as well as the lack of reduction in nonspecific transmittance indicates successful intercalation of BSA into the cn-CNC film and an absence of large protein domains, which would scatter light. Furthermore, the unchanged nonspecific transmittance of the M-BSA-25 spectrum indicates that BSA did not induce a strong aggregation when mixed with CNCs, favoring a homogeneous intercalation.

Coomassie blue staining was used to further assess protein infiltration in cn-CNC films. In acidic conditions, the red form of the dye is shifted to blue, Coomassie blue, e.g., if bound to proteins. Coomassie blue forms a strong, noncovalent complex with the carboxyl and amino groups of proteins^59^ and can be used to visualize protein distribution within material cross sections.^65^ Moreover, the staining solution fixes the protein in a material by denaturing it. Figure 4C shows microscope images of films, where the same position of the cross section was imaged in both reflection (left) and transmission (right) modes. A BSA-infiltrated CNC film was stained with Coomassie blue solution, yielding a dark-blue film. In the transmission microscopy image, the blue-colored dye can be observed across the thickness of the film, further inferring successful and homogeneous infiltration of proteins, from the top to the bottom of the film. As no gradient in blue color intensity is observed across the infiltrated CNC film, it can be concluded that the equilibration time of 24 h during the infiltration procedure is long enough to allow the protein to diffuse through the whole film and reach a constant concentration throughout the whole film. This observation is in accordance with literature reports on the protein infiltration of other materials.^65,66^ In contrast to the BSA-infiltrated CNC sample, a Coomassie-stained reference CNC film (I-REF-CNC) had a substantially reduced blue intensity.

Silk sericin (SS) and fibroin (SF), both structural proteins originating from silkworm cocoons, were infiltrated in cn-CNC films at concentrations < 4% (40 g L^–1^), e.g., to prevent protein agglomeration and precipitation. For the SS-infiltrated CNC film, I-SS-25 (Figure 5A), a substantial red shift in reflection was observed. A shift from transparent (I-REF-CNC, Figure 5A) to blue (I-SS-25, Figure 5B) can also be observed. Interestingly, the red shift did not increase at greater SS-to-CNC ratios (Figure S6A). The red shift caused by SS was also larger than for BSA (I-BSA-25 in Figure 4A). The lack of progressive helix stretching upon increased addition of SS is still unclear. Specific morphological features or shape-factors associated with the protein might explain the difference in infiltration, as imposed by the geometry of the interstices generated in the swollen cn-CNC films or the gelled, self-assembled films. The difference in the shape of SS (fibrillar) compared with BSA (globular) could favor intercalation across or within nematic pseudo-planes at high SS-to-CNC weight ratios.^67^ Possibly, a porous structure was formed upon redrying in the presence of SS, leading to a similar periodicity between I-SS-10 and I-SS-40, albeit with a difference in density for the latter.

A cn-CNC film obtained from a premixed SS solution and CNC suspension (M-SS-25) followed the trend observed for the reference samples I-REF-CNC and REF-CNC (Figure 5A), as was also the case for M-BSA-25 (Figure 4A). The exact localization of the proteins in the nanocomposites remains an open subject, especially in the premixed case. To fully understand the localization of each component, one would have to consider depletion effects as well as phase separation driving forces. Furthermore, there may be some impact from the macromolecules on evaporative flux inhomogeneities.^68^ In contrast, in the case of infiltration, the localization of the proteins would be principally dependent on the structure of the percolated network of CNCs in the stretched cn domains.

In the case of SF infiltration, a larger red shift in reflection was observed compared to both the BSA and SS infiltrations (Figure 5A). Similarly, there was a shift from transparent to blue, shown in Figure 5B. Also, in contrast to the SS infiltration, the red shift increased when increasing the fraction of SF, from 40 to 100, infiltrated into cn-CNC films (Figure S6B). Compared with SS, a large reduction in nonspecific transmission was observed, suggesting an increase in opacity, potentially from the formation of protein domains (Figure S6B). Note that infiltration is the only possibility to preserve the cn structure when creating composite films of CNCs and SF. Mixing CNCs and SF in suspension induces significant gelation (Figure S3). As previously shown,^39^ early gelation limits self-assembly of cn-CNC films.

Silk fibroin is of particular interest due to its ability to form disordered structures in chaotropic acidic conditions,^69,70^ which can be tuned into well-ordered β-sheet crystallites upon exposure to methanol or other alcohols. For this reason, we evaluated two additional polymorphs of silk fibroin. The first one, with increased number of β-sheets, was introduced in this work upon methanol treatment of a cn-CNC film infiltrated with SF (I-SF-25-β, Figure S7). The second one was obtained by denaturation of SF by formic acid prior to the infiltration (I-SF-25-denat). Both morphologies play a key role in defining the strength and rigidity of silk fibroin assemblies.^71−74^ In comparison to I-SF-25, the transmission spectrum of I-SF-25-β indicates a reduced red shift as well as a higher transmittance at wavelengths > 350 nm (Figure 5A). The blue shift and reduction in nonspecific transmittance are speculated to result from the lower volume occupied by the partially crystallized SF within the cn domains, which could lead to smaller pitch sizes (blue-shifted reflection). The photo of I-SF-25-β in Figure 5B indicates a reduced red shift compared to I-SF-25. In contrast to the previous polymorph (I-SF-25-β), the other polymorph I-SF-25-denat markedly reduced the transmittance, down to ∼30% (Figure 5A). The low pH of formic acid (approx. 2.3) caused protonation of the amine groups of SF (PI ∼ 3.8^75,76^), which resulted in partial aggregation. When infiltrating CNC films, the low flowability of the SF solution (nearly gelled) may have limited the infiltration efficiency; instead, SF may have coated the side of the CNC film where infiltration was attempted. Infiltration in acidic conditions might also affect the CNC–CNC interactions because CNC films swell significantly less when the sulfate group is protonated.^77−79^ Thus, the reduced swelling might lead to a lower protein uptake and explain the loss in nonspecific transmittance, e.g., by the formation of a protein layer on top of the CNC film. Microscope images were obtained from the cn-CNC films infiltrated with SS, SF, SF-β, and SF-denat, which further show the differences in color and texture between the proteins and polymorphs (Figure S5C). When infiltrated, a very homogeneous texture without the presence of segregated cn structures is observed. Potentially, the proteins cross-linked the swollen and infiltrated cn structure and prevented collapse into its preswollen state upon redrying. This latter assumption is corroborated by the presence of a wide reflection bandwidth after infiltration with SF. Upon β-sheet formation, the larger cn domains reappeared, which suggests further collapse of the cn structures. This was also observed, but to a lower extent, in the case of the denatured films.

Coomassie staining was carried out for cn-CNC films infiltrated with SS and SF (Figure 5D). The stained I-SS-25 and I-SF-25 films appeared dark blue across the whole cross section, especially in the transmission mode, implying homogeneous infiltration of protein through the whole thickness of the film.

**Figure 6:**
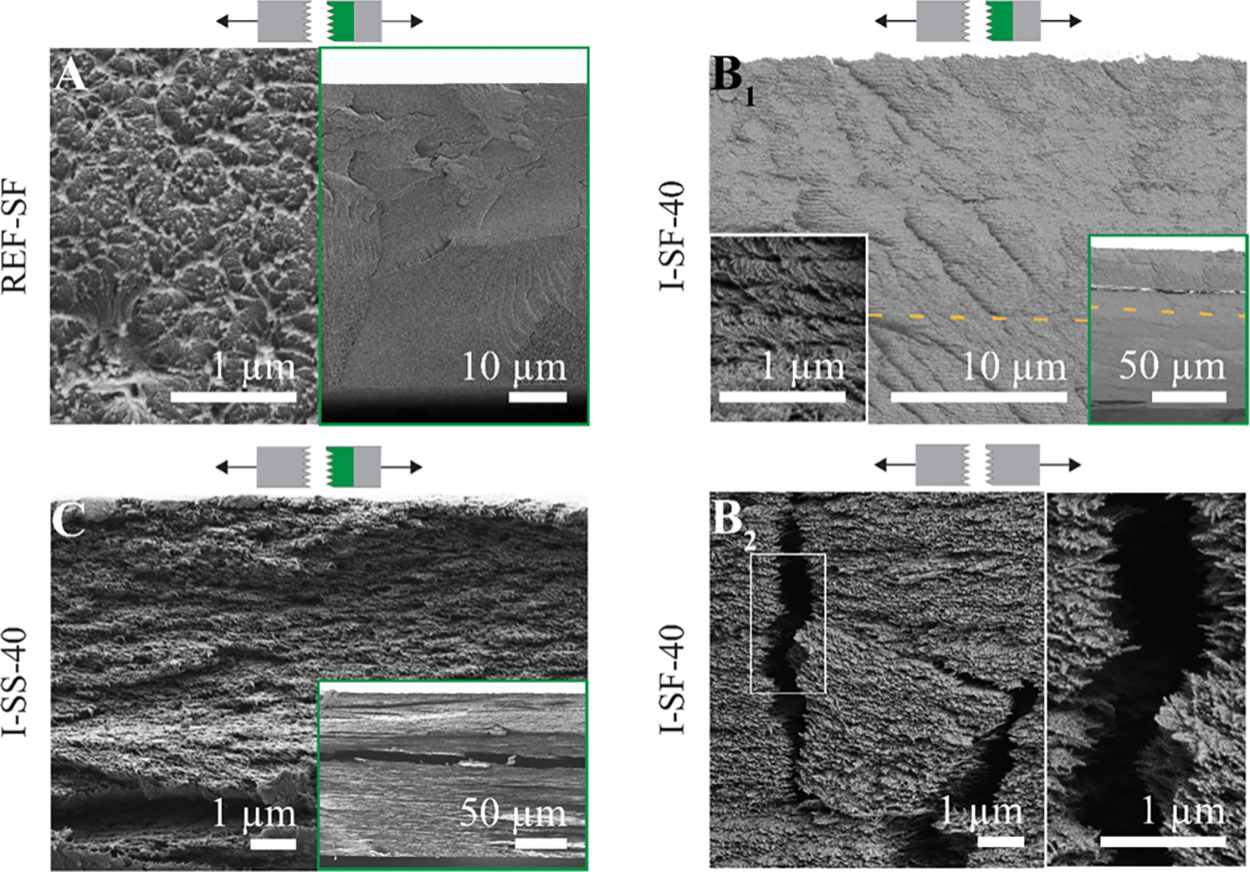
Scanning electron microscopy (SEM) images of silk fibroin (SF) films and CNC films infiltrated with SF and silk sericin (SS). The images show film cross sections fractured by tensile pulling, with a viewing direction along the film plane. Green framed insets show the full cross sections of films. (A) SF film. (B_1_, B_2_) CNC film infiltrated with SF. The white-framed inset in (B_1_) is a magnification from the cross section, and the (B_2_) inset is a magnification of the white box. (C) CNC film infiltrated with SS. The dashed orange lines in (B_1_) indicate the regions of images that were placed together.

The SS- and SF-infiltrated films were evaluated by SEM micrographs using the tensile fracture method, shown previously in Figures 3 and S5. The fracture of the pure SF sample was very sharp (Figure 6A right), indicating the brittle nature of the dry film. Ductile, nanometer-scaled domains were observed (Figure 6A left), as seen in the form of elevated features with a size of ca. 200 nm. The morphology of the internal structure “stretched” in the ductile domains, indicating the fibrillar form of the internal assembly. When observing the SF- and SS-infiltrated CNC films (Figure 6B,C), no protein domains were visible within the cross section or around the top or bottom parts of the film. Furthermore, the fractures were sharp, with completely conserved cn domains, indicating that the proteins interacted specifically with CNCs. Viscoelastic stretched domains could not be observed, even when exploring tortuous fractures where single nematic pseudo-layers can be isolated (Figure 6B_2_). These nano- to macro-scaled sharp fractures suggest the brittle nature of the composite films. Similar fractures were observed for the SS-infiltrated films (Figure 6C) although the “macro”-scaled fracture path (>100 nm fracture ridges) was slightly more tortuous for SS than for SF.

### Mechanical Properties of cn-CNC Films

**Figure 7:**
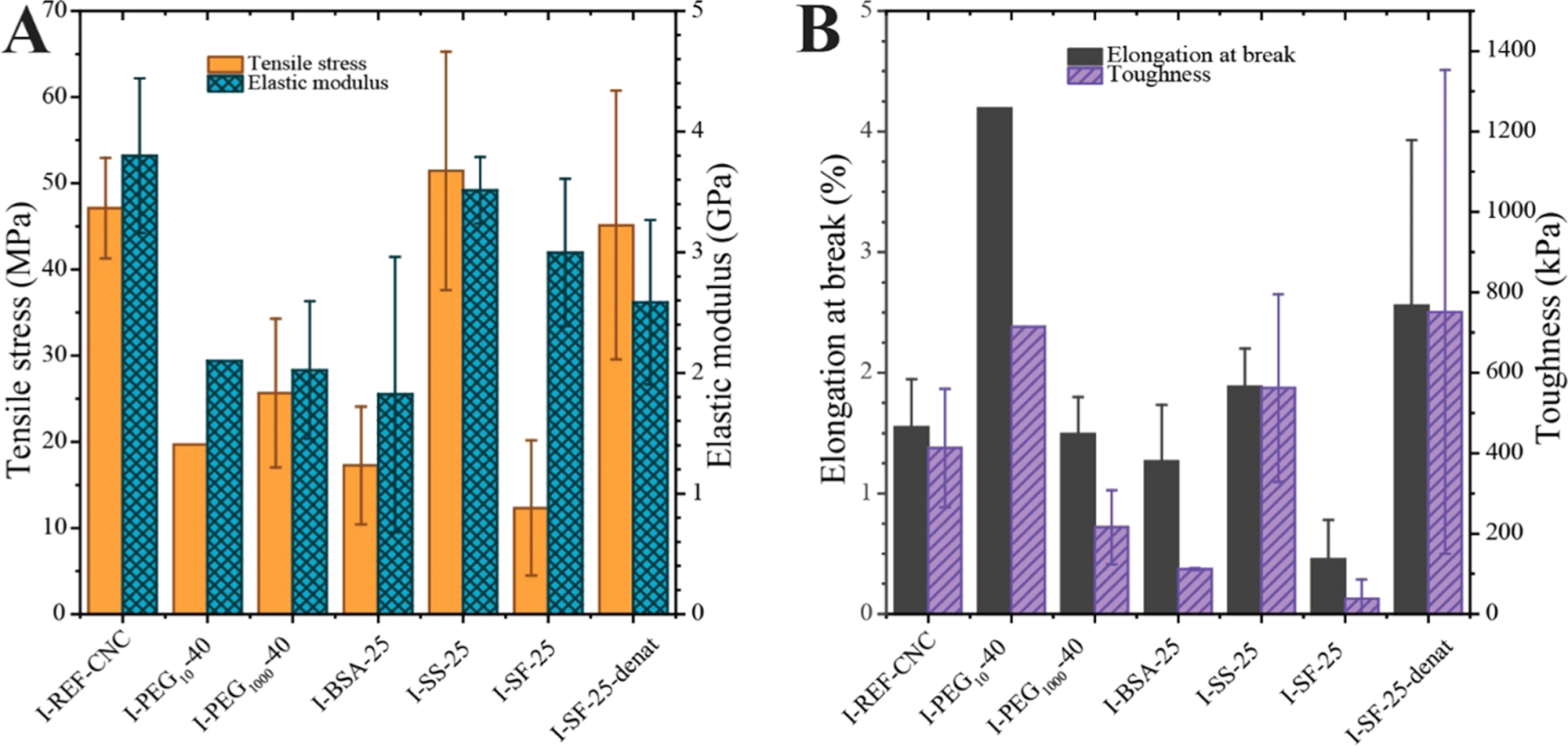
Mechanical properties of CNC films infiltrated with poly(ethylene glycol), PEG; bovine serum albumin, BSA; silk sericin, SS; and silk fibroin, SF. (A) Tensile stress and elastic modulus. (B) Elongation at break and toughness. Error bars represent standard deviation in measurement using five samples, except for I-PEG_10_-40 (*n* = 1), I-BSA-25 (*n* = 2), I-SS-25 (*n* = 2), and I-SF-25 (*n* = 3) that are shown for semiquantitative purposes.

We evaluated the material properties of the samples using tensile tests, as previously described,^21,31^ to test the influence of macromolecular additives on mechanical strength. Figure 7 shows a selection of mechanical properties of CNC films infiltrated with PEG, BSA, SS, and SF, obtained after conditioning them for 5 days (55 ± 3% RH, 23 °C), to make the CNC films easier to cut.^80^ Tensile data of all samples are summarized in Table S1. Note that solvent-cast protein films (BSA, SF, and SS) were too brittle to allow measurement.

The addition of PEG_10_ and PEG_100_ at a 100:40 CNC/PEG ratio increased, by 3-fold, the elongation at break of the infiltrated CNC films. The toughness increased by 1.4–1.7-fold, while the tensile stress of the material decreased by 1.9–3.1-fold (Figure 7). With increasing molecular weight of PEG, the elastic modulus did not significantly change and remained below the value measured for I-REF. As previously mentioned, PEG_1000_ could not fully penetrate the cn-CNC film, as observed in SEM images (Figure 3D). However, partial infiltration may have occurred, as suggested by the increased toughness and extension at break. In summary, through infiltration, PEG does have a plasticizing effect on CNC films, as previously reported for premixed systems,^32^ although it requires the MW to be < 400 kDa (Table S1).

For the BSA-infiltrated films, within the experimental error, the elongation at break did not significantly change compared with that of cn-CNC films. The stiffness and tensile strength decreased with an increased BSA ratio (Figures 7 and S8). Changing the protein structure from globular (BSA) to fibrillar (SS), while keeping the molecular weight and concentration fairly similar, led to an increased strength and stiffness, and an elongation that surpassed that of the CNC reference. Further, an increased silk sericin concentration resulted in a reduced strain at break and toughness of about 70% (Figure 7 and Table S1). The addition of silk fibroin, a fibrillar protein with an ∼5–23 times higher molecular weight than silk sericin, did not induce a significant difference in stiffness compared with CNC-REF, although the tensile strength and elongation decreased considerably. In contrast to BSA- and SS-infiltrated CNC films, the mechanical properties generally improved when the infiltration concentration of SF increased from 2.5 to 4%. The stiffness of I-SF-25-denat and I-SF-40-denat did not change with an increased ratio of denatured SF, whereas the toughness and elongation at break increased compared to I-SF-25, I-SF-40, and I-SS-40. The limiting factor affecting the mechanical properties could be the low apparent flowability of the 4 wt % solution of denatured SF in formic acid (a thick gel was observed, which led to deposition of protein on top of the CNC film, preventing full infiltration).

No segregated protein domains were identified by SEM and UV–vis, corroborating the successful formation of homogeneous composites. However, overall, the introduction of protein into the CNC matrix did not improve the cn-CNC mechanical properties despite the otherwise strong adhesion between the proteins and the CNCs.^81,82^ The reasons for this effect are still unclear and highlight the complex interactions between proteins and CNCs. For instance, several reports indicate gains in mechanical strength upon addition of silk fibroin to cellulose, with the latter in both the dissolved and fibrillated forms, although the strength of the cellulose component is generally weakened in such composites.^63,83−85^ There are a few examples of an enhancement of the mechanical properties of both components in cellulose–silk composite materials.^11,81,86^ Others studies indicate a negative influence of blending on material properties.^87^ These seemingly contradictory observations may relate to the homogeneity of the intercalation of the protein matrix, which otherwise forms strong materials. Likewise, the (tensile) properties of silk proteins, SF and SS, are strongly dependent on the method used for their extraction, e.g., using alkaline solution, steam, or ionic liquids.^88,89^ While the differences remain unclear, we herein provide a methodology for forming cn-CNC composites with SF, which cannot be achieved by other means.

## Conclusions

Aggregation of cellulose nanocrystals (CNCs) in the presence of strongly interacting building blocks is a limiting factor in the formation of chiral nematic (cn) CNC composite films. We investigate the infiltration of macromolecules into cn-CNC films to open the pathway to high-performance composites similar to those observed in nature. This approach enables the formation of cn nanocomposites based on macromolecules that could not be otherwise produced given the early gelation or strong aggregation (kinetic arrest). PEGs of various molecular weights, used as noninteracting model macromolecules, and strongly interacting macromolecules such as bovine serum albumin (BSA) and silk proteins were infiltrated while preserving the cn architectures, as evidenced by structural colors and SEM imaging. The structure of the swollen cn-CNC films enabled infiltration of macromolecules of up to 400 kDa, whereas PEG 1000 kDa formed a thick layer on top of the CNC film. The flowability was essential for infiltration, and 400 kDa PEG infiltrated better at a lower concentration, given the better flowability. All three proteins infiltrated readily and resulted in cn composite materials with a homogeneous distribution of the protein in the cn-CNC films, as evidenced by Coomassie staining of films. A denatured and more viscous form of SF did not infiltrate as efficiently when compared to nondenatured SF. Infiltration of PEG resulted in structures and mechanical properties comparable to those of films cast from a premixed PEG-CNC system. Despite the anticipated strong interaction between the CNCs and the silk proteins, no improvement in toughness or strength was observed in the corresponding films. Interestingly, a considerably higher toughness was observed for cn-CNC films infiltrated with the denatured SF, compared to the folded one. Furthermore, as we highlight in the discussion part when comparing performance with other cellulosic systems, there is a vast disparity in the observed synergism between cellulose and silk proteins. This suggests the need for a deeper understanding of their interfacial interactions and their cohesion as a material.

With the ever-increasing need to use biopolymers in the development of sustainable materials, this work considers a simple method to integrate such molecules in structured CNCs. Considering possible applications, macromolecule-infiltrated CNC materials offer a great platform for materials with functional properties, including colorimetric edible films, or biopolymeric films with controlled degradation. Chiral nematic nanocomposites with a range of macromolecules are expected to significantly enhance the compositional versatility. In the long-term perspective, we introduce a step toward engineered bionanocomposites.

## Supporting Information Available

Protocol to extract silk fibroin and silk sericin; protocol to stain protein-infiltrated CNC films with Coomassie blue; alternative protocol to prepare CNC films for Figure S2; additional SEM image of a cross section of a CNC film (Figure S1); microscope images and additional UV–vis transmission spectra of CNC reference films before and after swelling in water (Figure S2); photograph of gelled SF-CNC suspension (Figure S3); photographs of and additional UV–vis spectra of premixed PEG-CNC films (Figure S4); additional SEM images of cross sections and of PEG-infiltrated CNC films (Figure S5); additional UV–vis transmission spectra (SS, SF, Figure S6); FT-IR spectra of I-SF films (Figure S7); summary of tensile data of CNC–PEG and CNC–protein composites (Table S1 and Figure S8) (PDF)
