# Supplementary material for: Infiltration of Proteins in Cholesteric Cellulose Structures
Source: Biomacromolecules. 2021 Apr 26;22(5):2067–80. doi: 10.1021/acs.biomac.1c00183 (PMC8154265; doi:10.1021/acs.biomac.1c00183)
Supplement: Supplementary file 1 — bm1c00183_si_002.pdf [file bm1c00183_si_002.pdf]

Supporting Information

Infiltration of Proteins in Cholesteric Cellulose Structures

Livia K. Bast[†,‡], Konrad W. Klockars[§], Luiz G. Greca[§], Orlando J. Rojas[*,§,‖], Blaise L. Tardy[*,§], and Nico Bruns[*,†,‡]

[†]Adolphe Merkle Institute, University of Fribourg, Chemin des Verdiers 4, 1700 Fribourg, Switzerland.

[‡]Department of Pure and Applied Chemistry, University of Strathclyde, 295 Cathedral Street, Glasgow, G1 1XL, United Kingdom.

[§]Department of Bioproducts and Biosystems, School of Chemical Engineering, Aalto University, P.O. Box 16300, 00076 Aalto, Finland.

[‖]Departments of Chemical and Biological Engineering, Chemistry, and Wood Science, University of British Columbia, 2360 East Mall, Vancouver, British Columbia V6T 1Z4, Canada.

*Email: orlando.rojas@aalto.fi.

*Email: blaise.tardy@aalto.fi.

*Email: nico.bruns@strath.ac.uk.

L.K.B. and K.W.K contributed equally to this work.

Extraction of Silk Fibroin and Silk Sericin

Based on the standard protocol of Rockwood et. al.[1], silk fibroin was extracted from dried silk cocoons. In brief, silk cocoons were cut into pieces, the pupa was disposed, and fibers were degummed in a boiling solution of 0.02 M Na_2CO_3 for 2 h (5 g cocoon pieces per liter of respective solution). After degumming, the yellow solution containing silk sericin was kept until further use. Silk fibroin (SF) fibers were rinsed with water (5x15 min), dried in a fume hood overnight at room temperature and afterwards dissolved in Ajisawa's reagent[2] (molar ratio of $CaCl_2$:EtOH:H_2O = 1:3:8) at 65 °C, resulting in a 10 wt % solution of solubilized SF. Prior to use, the desired amount of SF solution was filtered (5 µm syringe filter, GE Health Care Whatman™) into a dialysis tube (Spectra/Por, MWCO 6-8 kDa, Spectrum Laboratories) and dialyzed against 1 L of Milli Q water for 3 days, exchanging the dialysis medium several times. The concentration of aqueous SF solution was determined by using an UV-Vis Spectrometer (NanoDrop™ ND-1000 Spectrophotometer) and Lambert-Beer's law (assuming a molecular weight of SF of 389,203 g mol^{-1}, based on ExPasy entry under the accession number P05790)[3], giving a concentration of 3 wt %. To concentrate SF solution further, dialysis was carried out against 10 wt % aqueous PEG (35 kDa) solution for 2-16 h, until the desired concentration was reached.

Silk sericin (SS) solution was lyophilized at -42 °C and at a pressure of > 0.2 mbar (TelStar LyoQuest Laboratory Freeze Dryer) and afterwards redissolved in ultrapure water. The solution was dialyzed against ultrapure water for 2 days (Spectra/Por dialysis membrane, MWCO 3.5 kDa, Spectrum Laboratories), exchanging the dialysis water several times. The concentration of SS solution was determined as mentioned above, assuming a molecular weight of SS of 117,276 g mol^{-1}, based on ExPasy entry under the accession number P07856).[3]

Coomassie Staining of Protein-Infiltrated CNC Films

In reference to Lämmli[4], a staining solution was prepared by dissolving 250 mg of Coomassie Brilliant Blue G-250 in 75 mL glacial acetic acid and 500 mL methanol. The dye solution was filled up to 1 L with ultrapure water and kept in an amber glass bottle to be protected from light. Destaining solution was prepared by mixing ultrapure water, glacial acetic acid and absolute ethanol in a ratio of 7:1:2 (V/V). Pieces of CNC films and protein-infiltrated CNC films were placed into separate petri dishes. The Petri dishes were filled up with Coomassie blue solution, covered and left overnight at ambient temperature. The dishes were lightly agitated every hour for the first four hours and the staining solution was discarded after 29 hours of staining. Destaining solution was added into the Petri dishes containing the stained films and discarded after a few minutes of agitation. This was repeated several times until no blue color was observed anymore in the used de-staining solution. During the staining/destaining procedures, no significant swelling was observed, and the films maintained their color as well as their mechanical integrity when in the staining/destaining solutions.

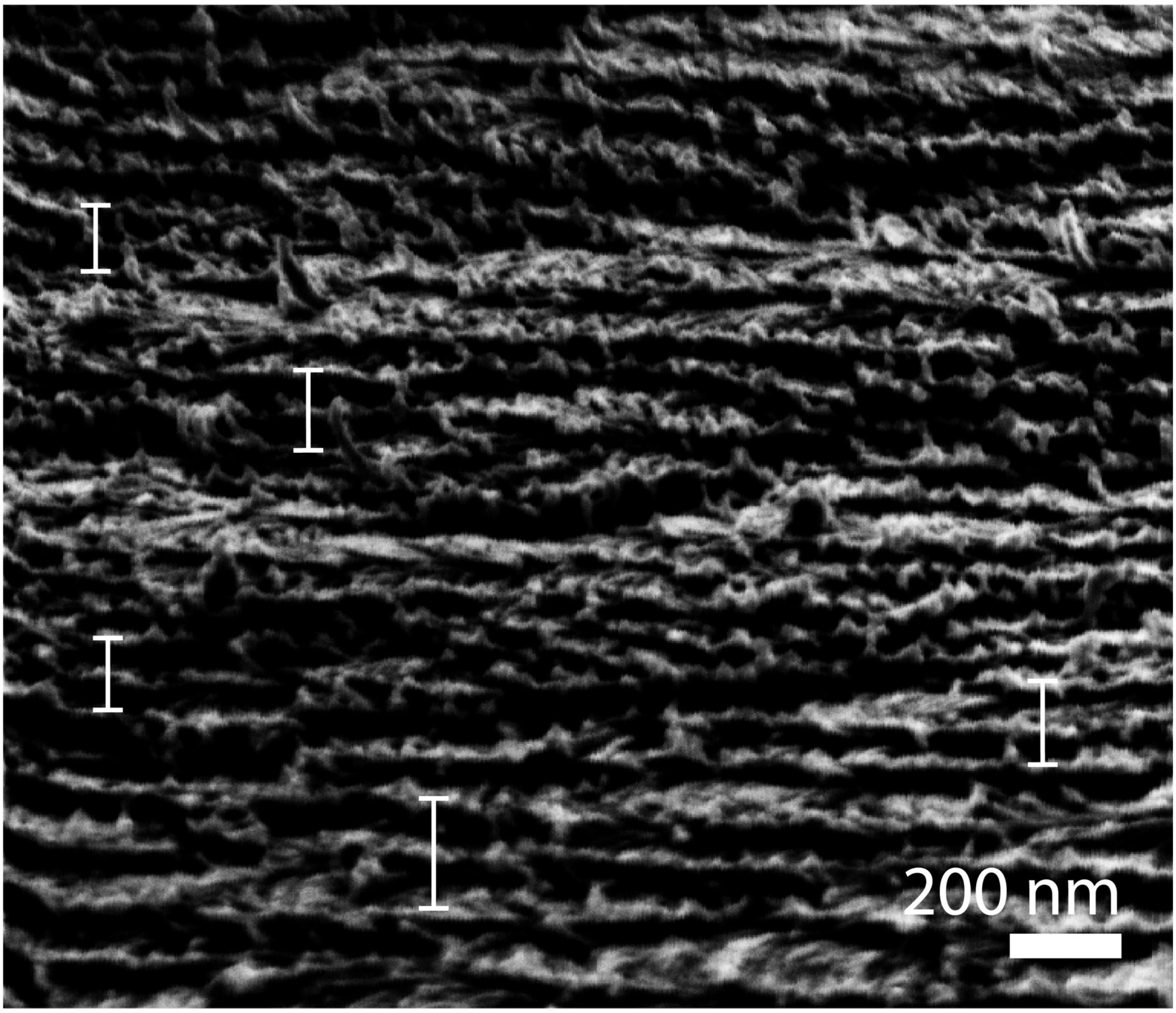

Figure S1. SEM image of the cross-section of a CNC film as evaluated for infiltration of macromolecules. The pitch size is measured from 5 different position, yielding an average value of 156 nm (standard deviation 27 nm). In a previous study,[5] the pitch size was measured from a CNC film that was cast from the same suspension that was used to produce CNC films for Figure S2 in this work. The calculated pitch value of those films was 440 nm, from six measurements of an SEM image (Figure4b3 in that work).

Alternative CNC Films

The CNC films used for Figure S2 were cast from a CNC suspension obtained from the same producer that provided the CNC suspensions for the rest of this work, but the batch was different, yielding different characteristics for the CNCs. Further information on characterization data of this batch can be found in our previous work.[5] The stock CNC suspension was diluted with Milli-Q water (Millipore, Synergy UV) to 5.5 wt %. 2 mL of suspension was poured into a 3.4 cm Petri dish, and a cn-CNC film was obtained through EISA in 50% relative humidity and 23°C. The film was then infiltrated with Milli-Q water, according to the infiltration procedure in the main text (section: "Infiltration of CNC films with Poly (ethylene glycol) and Proteins"). UV-Vis spectra were measured from the film before and after the infiltration step, from four different positions with a UV-vis-near-infrared (NIR) spectrophotometer (Agilent Cary 5000) in transmission mode. The film was also imaged in an Olympus BX53M optical microscope, in reflection mode, before and after infiltration.

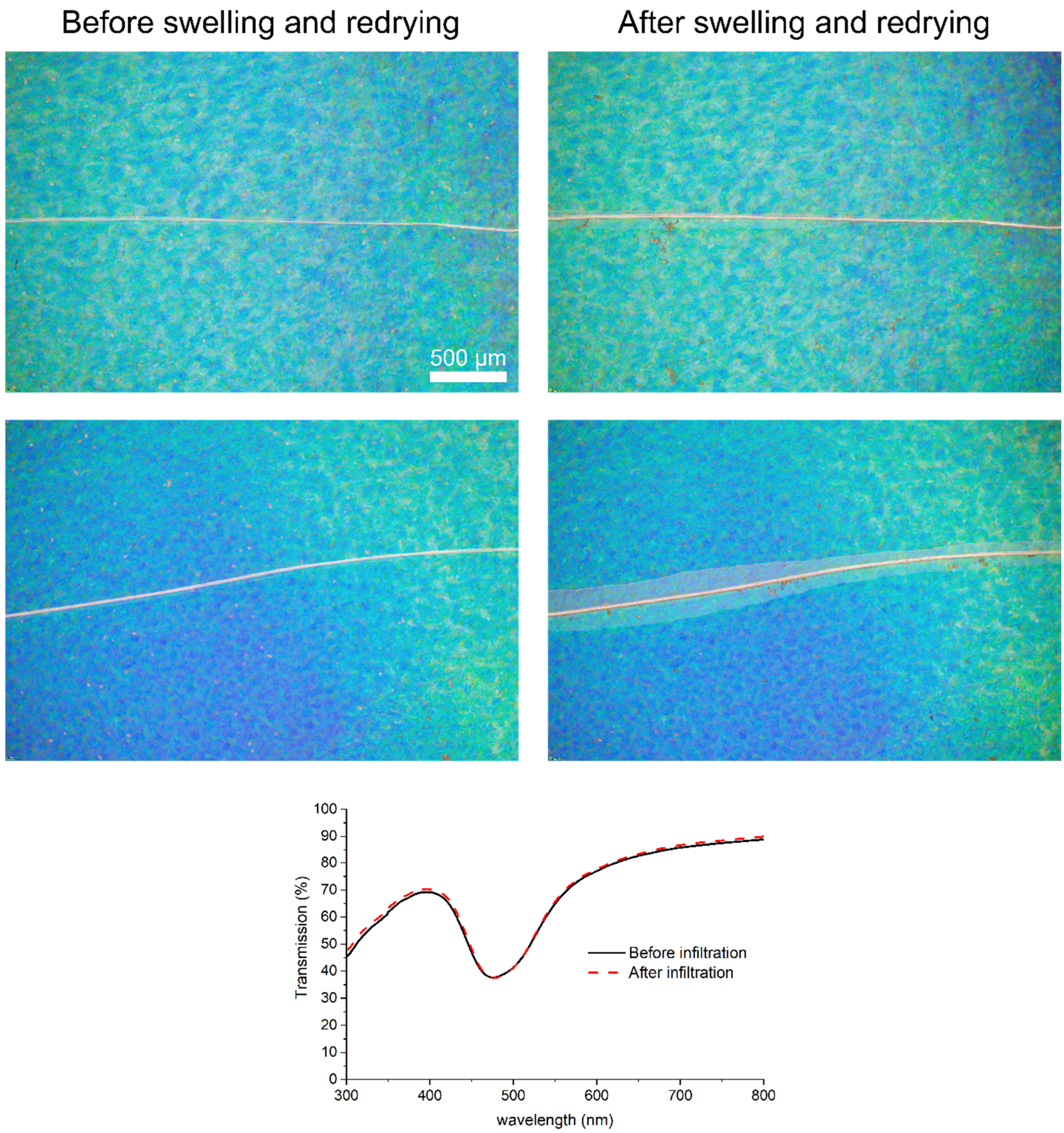

Figure S2. Microscope images and UV-vis spectra, showing the negligible effect on the cn order of swelling a pristine CNC film with water and subsequently drying it. Microscope images taken in reflection mode of the exact same positions (on each row) of a CNC film prior to swelling (left

column) and after swelling (right column). The scalebar applies for all images. UV-vis spectra of the same CNC film as shown in the microscopy images, before and after swelling and redrying (= infiltration).

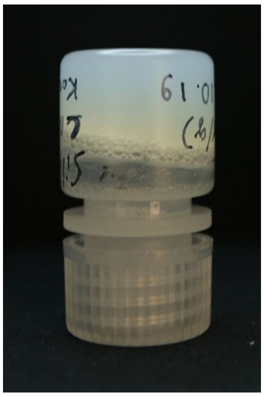

Figure S3. Photograph of fully gelled suspension containing 5.5 wt % CNC and 2.2 wt % SF. The photo is taken 2 days after turning the container upside down.

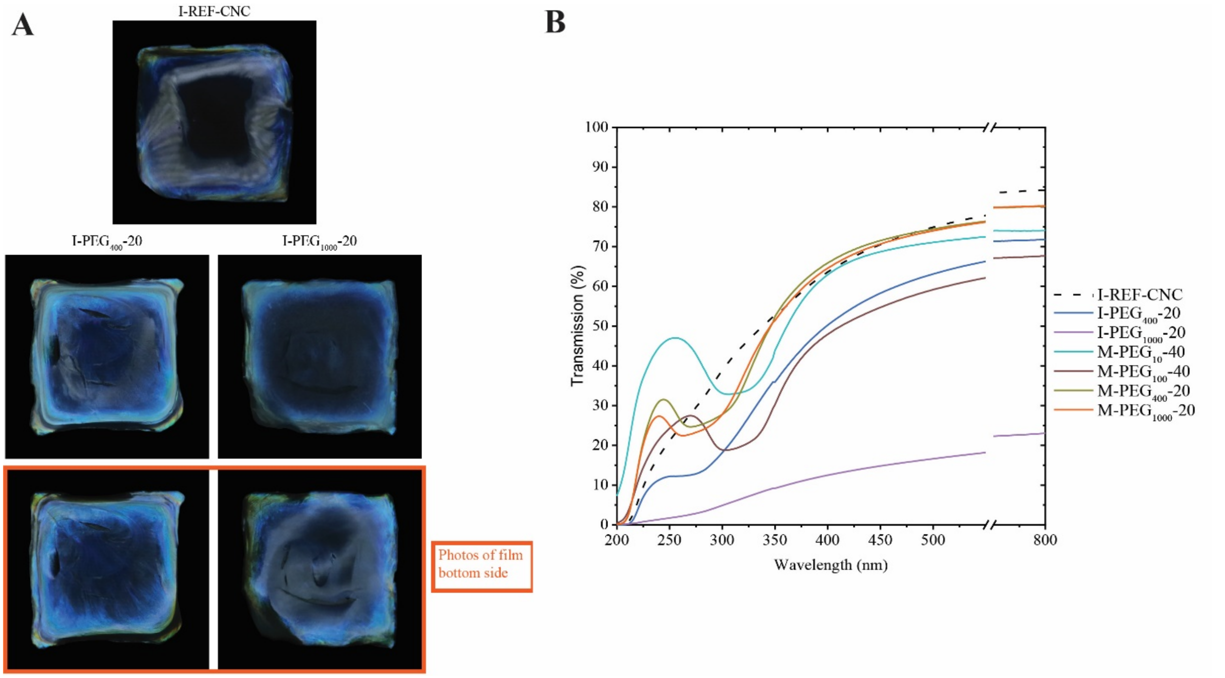

Figure S4. (A) Photographs of a CNC film infiltrated with water (top image) and CNC films infiltrated with PEG, taken normal to the film plane and parallel to the light source. Photos are taken from the top side, where the infiltrating solution was added. The photos within the orange box are taken from the bottom, non-infiltrated side. They are mirrored to facilitate easier comparison. (B) UV-Vis transmission spectra of CNC films infiltrated with PEG. The abscissa (x-axis) was broken in the 550-750 nm region to emphasize the redshifts below 550 nm.

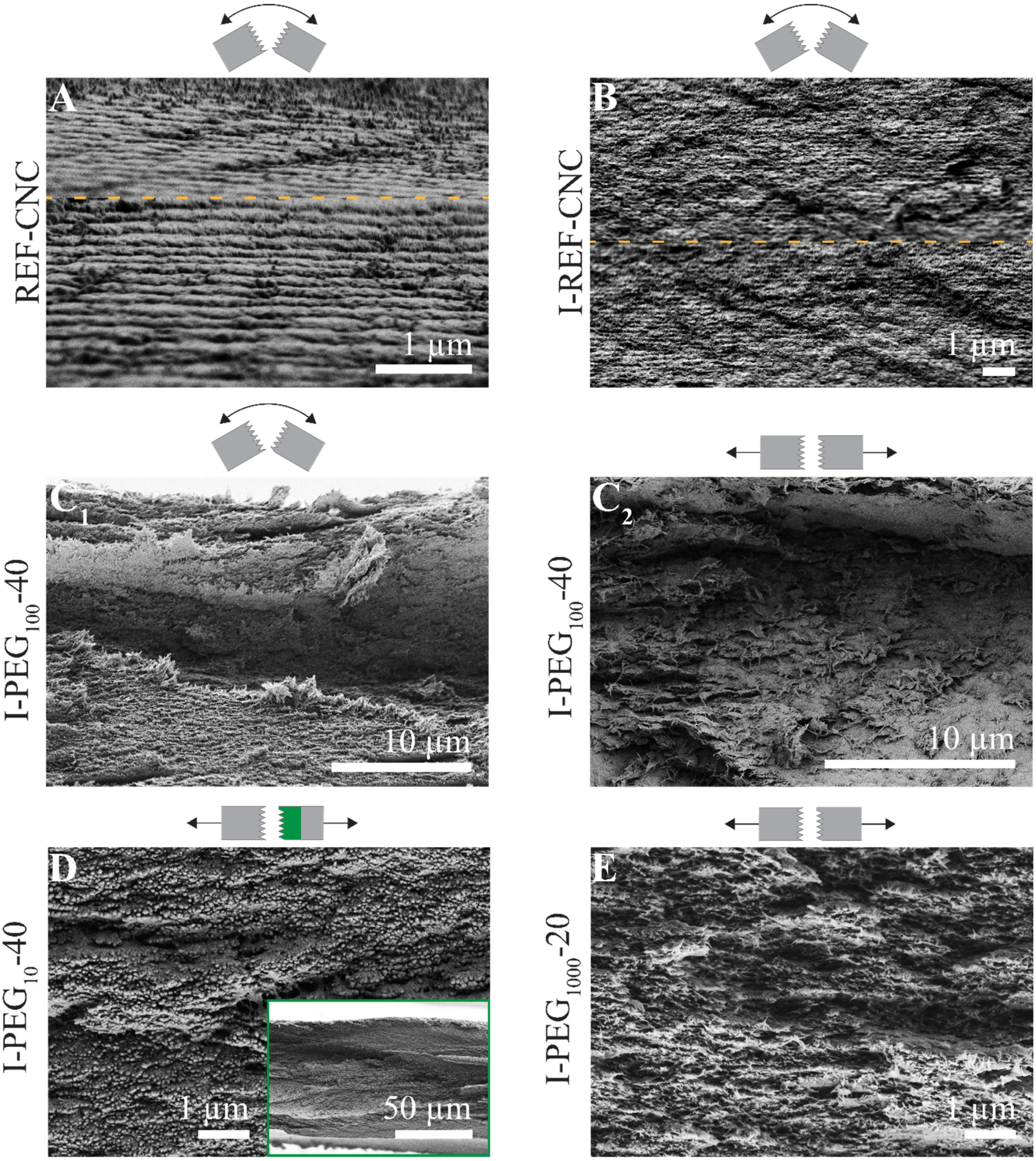

Figure S5. Scanning electron microscopy (SEM) images of CNC films infiltrated with water and poly (ethylene glycol) (PEG) of different molecular weights. A), (B) and (C_1) are cross-section images of films fractured by tensile pulling, where the viewing direction is along the film plane. (C_2), (D) and (E) are cross-section images of films fractured by bending, where the viewing

direction is normal to the film plane. (A) Crude CNC film prepared by EISA. (B) CNC film infiltrated (=swollen) with water and dried afterwards. ($C_{1,2}$) CNC film infiltrated with 100 kDa PEG. (D) CNC film infiltrated with 10 kDa PEG, and (E) CNC film infiltrated with 1000 kDa PEG. The dashed yellow lines indicate where separate images have been placed together, to allow for sharp focus despite a tilted surface. The images in the purple and blue frames are taken from the top and bottom parts of the film, respectively. Green framed insets show the full cross-sections of films.

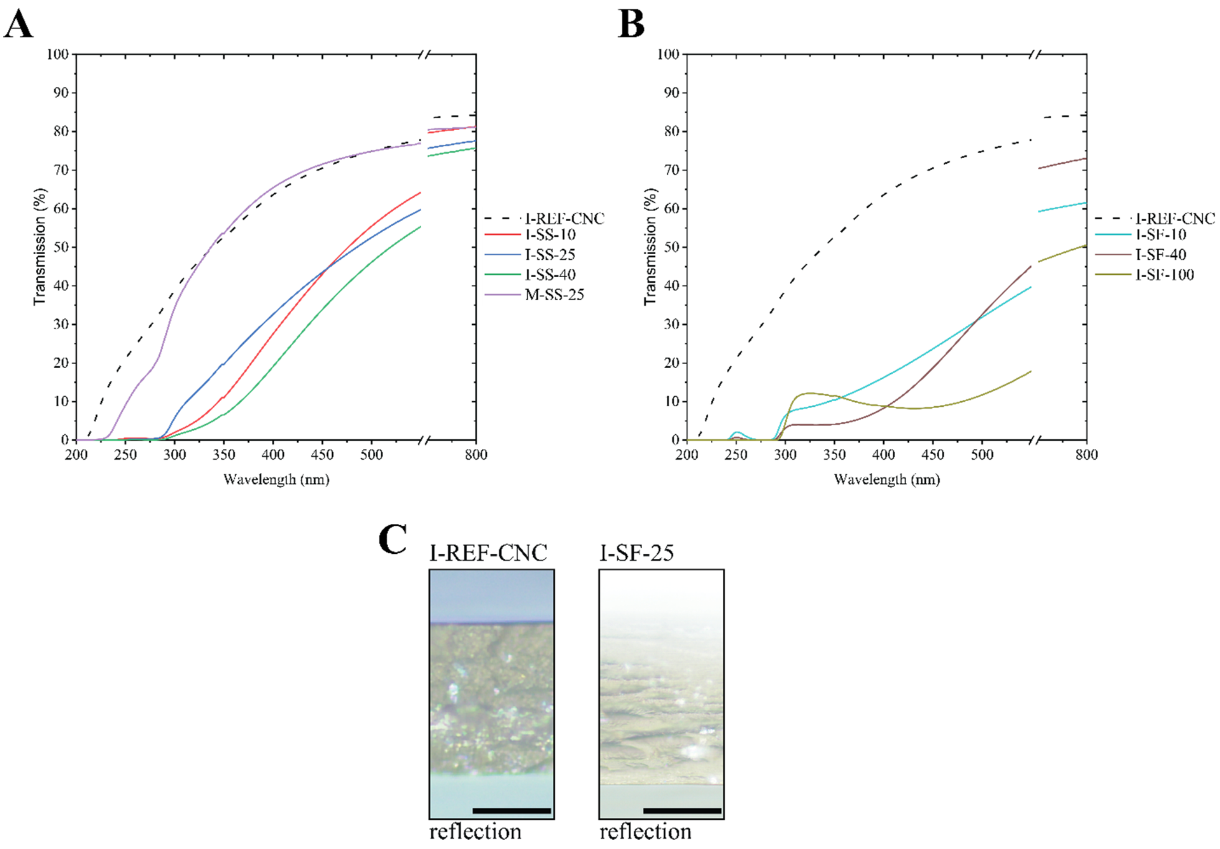

Figure S6. UV-vis transmission spectra of CNC films infiltrated with (A) silk sericin (SS) and (B) silk fibroin (SF). Some data is identical to that presented in Figure 5 to facilitate easy comparison.

The abscissa (x-axis) was broken in the 550-750 nm region to emphasize the redshifts below 550 nm. (C) Microscopy images taken in reflection mode of cross-sections of cn-CNC films, as unstained references for Figures 5C and 5D. The camera photodetectors were saturated when the cross-sections were imaged in transmission mode, under the same illumination conditions used for the microscopy images taken in transmission in Figures 5C and 5D. Therefore, the transmission images (I-REF-CNC and I-SF-25) are not shown. Please note that the bright areas in the reflection images of all samples are light reflections on the uneven surface of the cross sections. Scale bars correspond to 50 μm.

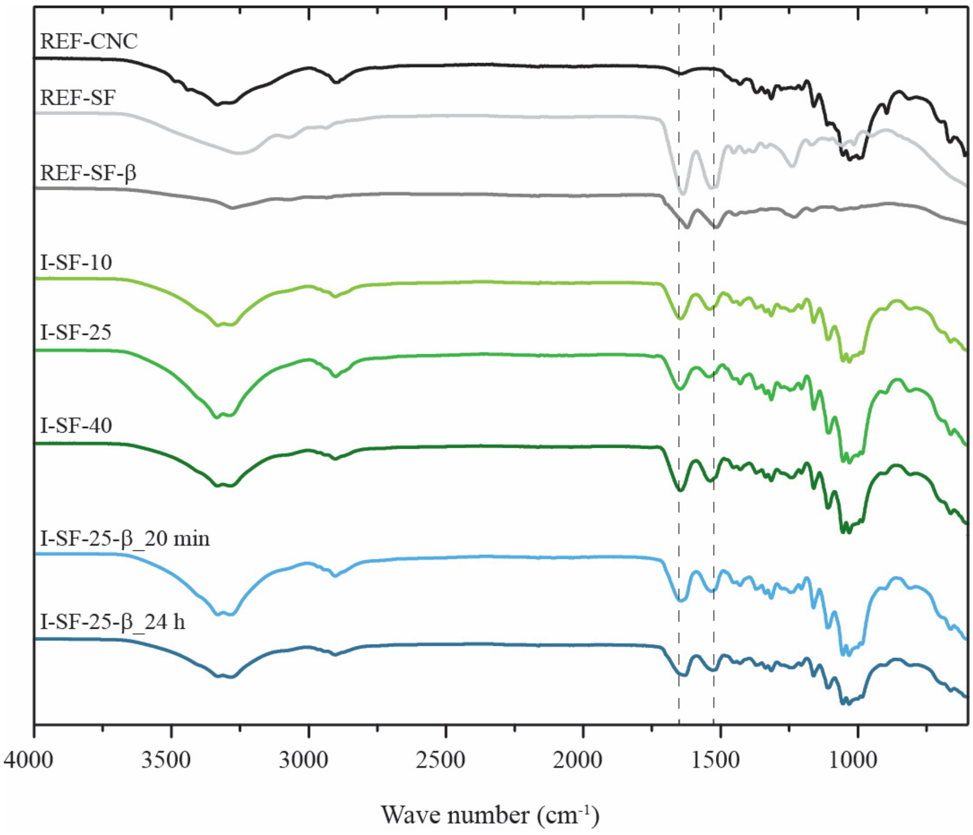

Figure S7. FT-IR spectra of I-SF films at different infiltration ratios before and after methanol treatment (20 min and 24 h, top to bottom). Reference spectra are shown, REF-CNC as dry CNC film, REF-SF as pure SF film and REF-SF-β as MeOH-treated SF film.

Table S1. Summary of Tensile Data of CNC-PEG and CNC-Protein Composites. The standard deviation was calculated from an average of five samples, except for I-PEG$_{10}$-40 (n = 1), I-BSA-25 (n = 2), I-BSA-50 (n = 1), I-SS-25 (n = 2) and I-SF-25 (n = 3).

sample code	*tensile stress, MPa*	*elastic modulus, GPa*	*elongation at break, %*	*toughness, kPa*
I-REF-CNC	47.1 ± 5.8	3.8 ± 6.0	1.6 ± 0.4	413 ± 147
I-PEG$_{10}$-40	19.7	2.1	4.1	714
I-PEG$_{100}$-40	15.3 ± 3.3	1.7 ± 0.5	4.4 ± 1.0	598 ± 240
I-PEG$_{400}$-20	10.3 ± 1.7	1.5 ± 0.5	1.6 ± 0.3	119 ± 31
I-PEG$_{1000}$-20	30.7 ± 2.8	3.6 ± 0.7	3.0 ± 0.5	741 ± 150
I-PEG$_{1000}$-40	25.7 ± 8.6	2.0 ± 0.6	1.5 ± 0.3	216 ± 93
I-BSA-10	22.4 ± 13.5	2.3 ± 0.9	1.1 ± 0.6	151 ± 144
I-BSA-25	17.3 ± 6.8	1.8 ± 1.1	1.3 ± 0.5	112 ± 3
I-BSA-50	6.2	1.1	0.7	21
I-SS-25	51.5 ± 13.8	3.4 ± 0.3	1.9 ± 0.3	562 ± 233
I-SS-40	29.6 ± 11.0	4.9 ± 0.8	0.6 ± 0.3	103 ± 91
REF-SF[a,6–8]	10-25	0.7-2.5	~ 1.0-2.4	N/A
I-SF-25	12.3 ± 7.9	3.0 ± 0.6	0.5 ± 0.3	38 ± 47
I-SF-40	23.3 ± 8.6	4.9 ± 1.6	0.5 ± 0.3	68 ± 38
I-SF-25-denat	45.1 ± 15.6	2.6 ± 0.7	2.6 ± 1.4	751 ± 601

[a]No numerical values given in Noishiki *et al.* and Freddi *et al.*[6,7], data presented here are taken from graphs of tensile data. In the latter publication, the degumming procedures for the extraction

of silk fibroin from the cocoon varied to our procedure which also has an impact on the mechanical properties of silk fibroin.

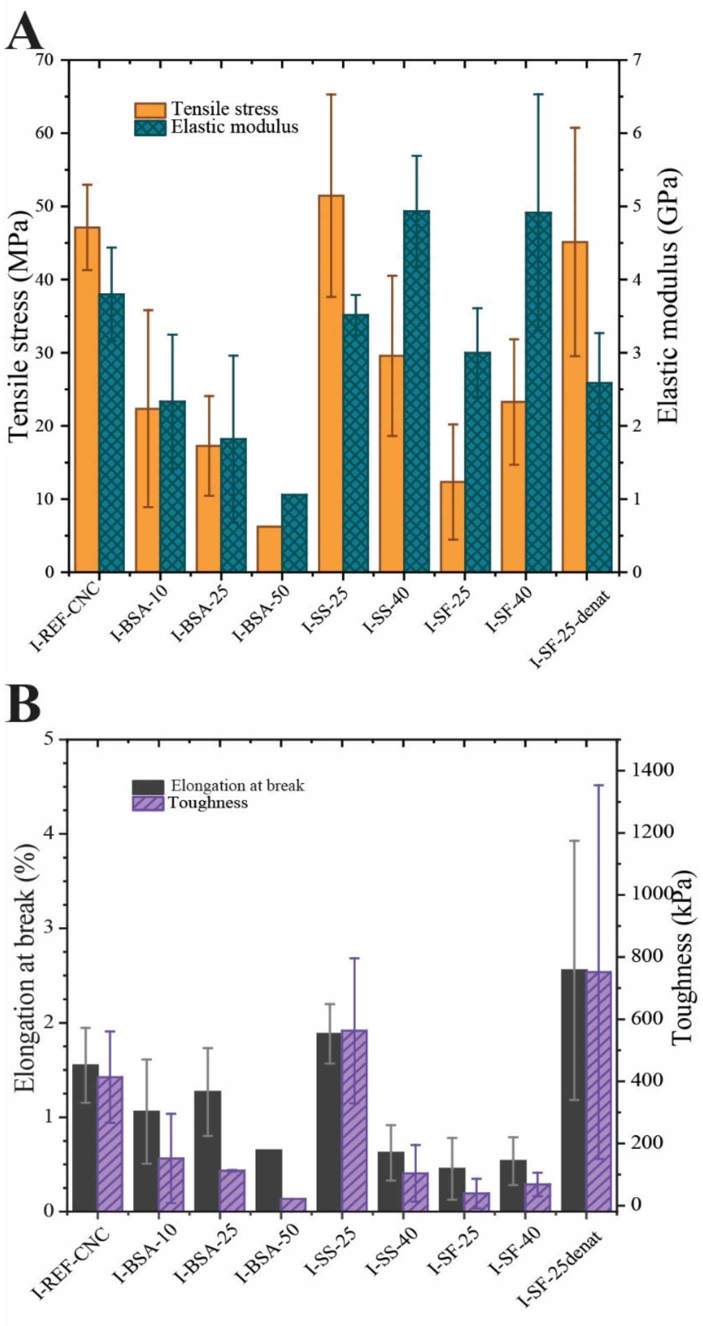

Figure S8. Tensile data of all protein-infiltrated CNC films. (A) Tensile stress and elastic modulus, (B) elongation at break and toughness. Error bars represent standard deviation of five samples,

except for I-BSA-25 (n = 2), I-BSA-50 (n = 1), I-SS-25 (n = 2), and I-SF-25 (n = 3). Some of the data presented here were compiled into Figure 7 to facilitate easy comparison.

References

(1) Rockwood, D. N.; Preda, R. C.; Yucel, T.; Wang, X.; Lovett, M. L.; Kaplan, D. L. Materials Fabrication from *Bombyx Mori* Silk Fibroin. *Nat. Protoc.* **2011**, *6*, 1612–1631. 10.1038/nprot.2011.379.

(2) Ajisawa, A. Dissolution of Silk Fibroin with Calciumchloride/Ethanol Aqueous Solution. The *Journal Sericultural Science of Japan* **1998**, *67*, 91–94. 10.11416/kontyushigen1930.67.91.

(3) Artimo, P.; Jonnalagedda, M.; Arnold, K.; Baratin, D.; Csardi, G.; de Castro, E.; Duvaud, S.; Flegel, V.; Fortier, A.; Gasteiger, E.; Grosdidier, A.; Hernandez, C.; Ioannidis, V.; Kuznetsov, D.; Liechti, R.; Moretti, S.; Mostaguir, K.; Redaschi, N.; Rossier, G.; Xenarios, I.; Stockinger, H. ExPASy: SIB Bioinformatics Resource Portal. *Nucleic Acids Res.* **2012**, *40*, W597–W603. 10.1093/nar/gks400.

(4) Lämmli, U. K. Cleavage of Structural Proteins during the Assembly of the Head of Bacteriophage T4. *Nature* **1970**, *227*, 680–685. 10.1038/227680a0.

(5) Klockars, K. W.; Tardy, B. L.; Borghei, M.; Tripathi, A.; Greca, L. G.; Rojas, O. J. Effect of Anisotropy of Cellulose Nanocrystal Suspensions on Stratification, Domain Structure Formation, and Structural Colors. *Biomacromolecules* **2018**, *19*, 2931–2943. 10.1021/acs.biomac.8b00497.

(6) Noishiki, Y.; Nishiyama, Y.; Wada, M.; Kuga, S.; Magoshi, J. Mechanical Properties of Silk Fibroin–Microcrystalline Cellulose Composite Films. *J. Appl. Polym. Sci.* **2002**, *86*, 3425–3429. 10.1002/app.11370.

(7) Freddi, G.; Romanò, M.; Massafra, M. R.; Tsukada, M. Silk Fibroin/Cellulose Blend Films: Preparation, Structure, and Physical Properties. *J. Appl. Polym. Sci.* **1995**, *56*, 1537–1545. 10.1002/app.1995.070561203.

(8) Kaewpirom, S.; Boonsang, S. Influence of Alcohol Treatments on Properties of Silk-Fibroin-Based Films for Highly Optically Transparent Coating Applications. *RSC Adv.* **2020**, *10*, 15913–15923. 10.1039/D0RA02634D.
